# A de novo genome assembly of *Solanum bulbocastanum* Dun., a Mexican diploid species reproductively isolated from the A-genome species, including cultivated potatoes

**DOI:** 10.1093/g3journal/jkae080

**Published:** 2024-04-12

**Authors:** Awie J Hosaka, Rena Sanetomo, Kazuyoshi Hosaka

**Affiliations:** Nihon BioData Corporation, Takatsu, Kawasaki, Kanagawa 213-0012, Japan; Kihara Institute for Biological Research, Yokohama City University, Yokohama 244-0813, Japan; Potato Germplasm Enhancement Laboratory, Obihiro University of Agriculture and Veterinary Medicine, Obihiro, Hokkaido 080-8555, Japan; Potato Germplasm Enhancement Laboratory, Obihiro University of Agriculture and Veterinary Medicine, Obihiro, Hokkaido 080-8555, Japan

**Keywords:** *Solanum bulbocastanum*, Mexican wild diploid potato, de novo genome assembly, comparative genomics

## Abstract

Potato and its wild relatives are distributed mainly in the Mexican highlands and central Andes of South America. The South American A-genome species, including cultivated potatoes, are reproductively isolated from Mexican diploid species. Whole-genome sequencing has disclosed genome structure and similarity, mostly in cultivated potatoes and their closely related species. In this study, we generated a chromosome-scale assembly of the genome of a Mexican diploid species, *Solanum bulbocastanum* Dun., using PacBio long-read sequencing, optical mapping, and Hi-C scaffolding technologies. The final sequence assembly consisted of 737.9 Mb, among which 647.0 Mb were anchored to the 12 chromosomes. Compared with chromosome-scale assemblies of *S. lycopersicum* (tomato), *S. etuberosum* (non-tuber-bearing species with E-genome), *S. verrucosum*, *S. chacoense*, *S. multidissectum*, and *S. phureja* (all four are A-genome species), the *S. bulbocastnum* genome was the shortest. It contained fewer transposable elements (56.2%) than A-genome species. A cluster analysis was performed based on pairwise ratios of syntenic regions among the seven chromosome-scale assemblies, showing that the A-genome species were first clustered as a distinct group. Then, this group was clustered with *S. bulbocastanum*. Sequence similarity in 1,624 single-copy orthologous gene groups among 36 *Solanum* species and clones separated *S. bulbocastanum* as a specific group, including other Mexican diploid species, from the A-genome species. Therefore, the *S. bulbocastanum* genome differs in genome structure and gene sequences from the A-genome species. These findings provide important insights into understanding and utilizing the genetic diversity of *S. bulbocastanum* and the other Mexican diploid species in potato breeding.

## Introduction

Modern potato cultivars (*Solanum tuberosum* L., 2*n* = 4*x* = 48), together with Andean native cultivars and over 100 closely related wild species, form section *Petota* in the genus *Solanum* (Hawkes 1990; Spooner *et al.* 2014). Hawkes (1990) further separated non-tuber-bearing and tuber-bearing species in the section *Petota* into subsections *Estolonifera* and *Potatoe*, respectively. Recent molecular phylogenetics supported the two subsections as Etuberosum and Petota clades, respectively, and, together with Tomato and some other minor clades, formed the large Potato clade (Gagnon *et al.* 2022). Their centers of diversity are found in the Mexican highlands and the central Andes (Hawkes 1990; Spooner *et al.* 2014). The diploid species of the two centers of diversity are strictly isolated from each other by reproductive barriers (Hawkes 1958). Based on the meiotic chromosome pairing of interspecific hybrids, the A-genome was assigned to the South American species, including cultivated potatoes (Matsubayashi 1991). These species are referred to as A-genome species hereafter. Since sexual hybrids between Mexican diploid species and A-genome species are extremely difficult to obtain, the genome affinity of Mexican diploid species has long been argued (Matsubayashi 1991; Pendinen *et al.* 2008).

Mexican diploid species are grouped into four taxonomic series (*Morelliformia*, *Bulbocastana*, *Pinnatisecta*, and *Polyadenia*) (Hawkes 1990). Meiotic chromosome behaviors in interspecific hybrids between Mexican diploid species from different taxonomic series indicated that their genomes are somewhat more diverged from each other than those between A-genome species from the South American series (Magoon *et al.* 1958; Marks 1968; Matsubayashi and Misoo 1977). Molecular analyses revealed clear genome differentiation of Mexican diploid species from the A-genome species (Hosaka *et al.* 1984; Spooner *et al.* 1991, 2008; Pendinen *et al.* 2008; Rodríguez and Spooner 2009). Recent advancement in nucleotide sequencing technologies allows comparing whole-genome sequences of the tuber-bearing *Solanum* species (Aversano *et al.* 2015; Leisner *et al.* 2018; Zhou *et al.* 2020; Tiwari *et al.* 2021; Yan *et al.* 2021; Hoopes *et al.* 2022; Hosaka *et al.* 2022; Sun *et al.* 2022), which revealed a monophyletic origin of Mexican diploid species sharing the common ancestor with A-genome species (Li *et al.* 2018; Huang *et al.* 2019; Gagnon *et al.* 2022; Tang *et al.* 2022).

*Solanum bulbocastanum* Dun. (2*n* = 2*x* = 24), a representative species of the series *Bulbocastana*, is morphologically distinct with simple leaves, without lateral leaflets, from most of the other *Petota* species and well known for its resistance to late blight (caused by *Phytophthora infestans*) and Columbia root-knot nematode (*Meloidogyne chitwoodi*) (Graham *et al.* 1959; Toxopeus 1964; Bali *et al.* 2022). The late blight resistance genes *Rpi-blb1/RB*, *Rpi-blb2*, *Rpi-blb3*, and *Rpi-bt1* have been cloned from this species (Song *et al.* 2003; Lokossou *et al.* 2010). Although direct crosses with cultivated potatoes are impossible, breeders transferred these resistance genes by bridging crosses via *S. verrucosum* Schlechtendal and by somatic hybridization (Hermsen and Ramanna 1976; Austin *et al.* 1993; Brown *et al.* 1995; Jansky and Hamernik 2009; Tiwari *et al.* 2018). Thus, *S. bulbocastanum* is an important species to investigate from genome evolution and potato breeding standpoint.

The *S. bulbocastanum* PG6241 genome has been sequenced to the level of contigs using a PacBio long-read sequencing technology (Tang *et al.* 2022). In this study, we generated a chromosome-scale assembly of the *S. bulbocastanum* genome using PacBio long-read sequencing and, in addition, Optical mapping and Hi-C scaffolding technologies. A naturally chromosome-doubled monohaploid clone of *S. bulbocastanum* (2*n* = 2*x* = 24) was used to reduce the complexity caused by the heterozygous nature of the species. This is the Mexican diploid species’ first de novo chromosome-scale assembly except for *S. verrucosum*, the only diploid A-genome species distributed in Mexico (Hosaka *et al.* 2022). The chromosome-scale assemblies facilitated to reveal structural differences between the *S. bulbocastanum* genome and the previously reported *S. phureja*, *S. multidissectum*, *S. verrucosum*, *S. chacoense*, *S. etuberosum*, and *S. lycopersicum* genomes (Leisner *et al.* 2018; Hosaka *et al.* 2022; Tang *et al.* 2022; Zhou *et al.* 2022; Yang *et al.* 2023) in the Potato, Etuberosum, and Tomato clades.

## Materials and methods

### Plant material

A naturally doubled monohaploid clone of *S. bulbocastanum* (11H21, available as PI 666967 from the US Potato Genebank at Sturgeon Bay, WI, USA) that was derived from anther culture (Irikura and Sakaguchi 1972) and maintained in vitro in our laboratory (Sanetomo and Hosaka 2021), was used for sequencing.

### DNA extraction, genome sequencing, and initial assembly

High-molecular-weight DNA was extracted, qualified, and used for HiFi read sequencing using the PacBio Sequel lle system (PacBio) as previously described (Hosaka *et al.* 2022). The bam files were converted to a FASTQ file using BAM2fastx 1.3.1 (PacBio) and used for genome assembly with the Hifiasm 0.16.1-r375 assembler (Cheng *et al.* 2021). The “-l 0” option was used to turn off purge haplotigs since the plant was a completely homozygous diploid clone.

### Optical mapping

Genomic DNA was extracted from young leaves using the Plant DNA Isolation Kit (Bionano Genomics, San Diego, CA, USA). The isolated DNA was labeled with Direct Labeling Enzyme 1 using the DLS DNA Labeling Kit (Bionano Genomics, San Diego, CA). The labeled DNA was loaded onto a Saphyr chip and run on the Saphyr Optical Genome Mapping (OGM) Instrument (Bionano Genomics). The output molecules were assembled and then merged with the contigs to generate hybrid scaffold sequences using OGM-specific pipelines Bionano Access and Solve (versions 1.7.1.1 and 3.7_03302022_283, respectively) with default parameters. AS ONE Corp. (Osaka, Japan) collected and analyzed data.

### Hi-C sequencing and scaffolding

To construct the Hi-C library, proximity ligation and library amplification were performed by using the Dovetail Omni-C Kit (Dovetail Genomics, Scotts Valley, CA, USA) and the Kapa Hyper Prep kit (KAPA Biosystems, Cape Town, South Africa), respectively. The sampled leaves were ground in liquid nitrogen, resuspended with 5 ml of PBS buffer with 1% formaldehyde, and rotated for 10 min. The sample was centrifuged at 5000*×g* for 5 min, and the supernatant was discarded. The precipitate was resuspended with 5 ml of Nuclei Isolation Buffer (10 mM HEPES, 5 mM KCl, 5 mM MgCl_2_, 5 mM EDTA, 1 M sucrose, and 0.2% Triton X-100). The sample was filtrated through a 40-μm cell strainer. The filtrated sample was centrifuged at 5000*×g* for 5 min, and the supernatant was discarded. The precipitate was resuspended with 1 ml of Nuclei Isolation Buffer, centrifuged at 5000*×g* for 5 min, and the supernatant was discarded. The residue was resuspended with 500 μl Nuclei Isolation Buffer and layered on 500 μl Nuclei Separation Buffer (10 mM HEPES, 5 mM KCl, 5 mM MgCl_2_, 5 mM EDTA, 1 M sucrose, and 15% Percoll). The sample was centrifuged at 3000*×g* for 5 min, and the supernatant was discarded. The precipitate was used for DNase treatment and proximity ligation steps. The ligation product was eluted with 50 μl of TE buffer (10 mM Tris–Cl buffer pH 8.0 and 1 mM EDTA pH 8.0). Ten microliter of Dynabeads MyOne Streptavidine C1 beads was rinsed in 150 μl of TWB buffer and resuspended with 100 μl of 2× NTB buffer. Fifty microliter of the ligation product and 50 μl of TE buffer were mixed with the beads and rotated for 15 min. The beads were washed once with 500 μl of LWB, twice with 500 μl of NWB, once with 200 μl of Wash Buffer, and resuspended with 25 μl of sterile water. The resuspended beads were mixed with 3.5 μl of End Repair & A-tailing Buffer and 1.5 μl of End Repair & A-tailing Enzyme Mix and incubated at 20 °C for 30 min and then, at 65 °C for 30 min. The reaction solution was mixed with 15 μl of Ligation Buffer, 5 μl of DNA Ligase, and 2.5 μl of Dual-index adapter and incubated at 20 °C for 15 min. The beads were washed once with 500 μl of LWB, twice with 500 μl of NWB, once with 200 μl of Wash Buffer, and resuspended with 10 μl of TE buffer. Then, 12 μl of 2× KAPA HiFi Hot Start Ready Mix and 2.5 μl of 2× Library Amplification Primer Mix were added to the solution. Polymerase chain reaction (PCR) was performed with the following conditions: 98 °C for 45 s, then 16 cycles of 98 °C for 15 s, 60 °C for 30 s, and 72 °C for 30 s, and a final extension at 72 °C for 1 min. The supernatant of the PCR product was purified and dual size-selected using 0.5× and 0.3× volumes of AMPure XP beads (Beckman, Indianapolis, IN, USA) and eluted with 12 μl of TE buffer.

The prepared library was sequenced on the NovaSeq 6000 (Illumina, San Diego, CA) platform. The reads were aligned to the scaffolded contigs using Juicer 1.6 (Durand *et al.* 2016). Since DNase I was used to digest fixed nucleosomes, the “-s none -y none” option was specified. The generated contact maps were then used for scaffolding with a 3D-DNA pipeline (Dudchenko *et al.* 2017) with the “–rounds 0” parameter. The scaffolds were reviewed and manually corrected using JuiceBox 1.11.08 (https://github.com/aidenlab/Juicebox). Identities and directions of the corrected scaffolds were determined based on the alignment to the *S. phureja* Juz. & Bukasov (described as *S. tuberosum* Andigenum group in Spooner *et al.* 2014) DM v8.1 reference genome (Yang *et al.* 2023) using RagTag (Alonge *et al.* 2022).

### Annotation

To construct homology-based gene models, annotation results of *S. bulbocastanum* PG6241 (Tang *et al.* 2022) were lifted to our assembly using Liftoff (Shumate and Salzberg 2021) with the “-polish -copies” option. To construct RNA-seq-based gene models, publicly available datasets of *S. bulbocastanum* PG6241 in Sequence Read Archive (SRA) database (SRR15560132, SRR15560142, SRR15560143, SRR15560145, SRR15560146, SRR15560147, SRR15560148, SRR15560149, SRR15560150, SRR15560151, SRR15560152, and SRR15560153) were obtained using Sratools (https://github.com/ncbi/sra-tools). After filtering the low-quality reads using Fastp 0.23.2 (Chen *et al.* 2018), the reads were aligned to the assembled genome using Hisat2 2.2.1 (Kim *et al.* 2019). Putative transcripts were identified in each *S. bulbocastanum* dataset, and the resulting General Transfer Format (GTF) files were merged using Stringtie 2.2.1 (Kovaka *et al.* 2019) and used to search putative open-reading-frames by TransDecoder.LongOrfs (Haas BJ, https://github.com/TransDecoder/TransDecoder) with default parameters. To improve the prediction accuracy of protein-coding regions, we performed Blastp search of the putative amino acids against *S. bulbocastanum* PG6241 protein sequences with the “-max_target_seqs 1 -outfmt 6 -evalue 1e-5” parameter. Protein-coding regions were predicted using TransDecoder.Predict with the Blastp result and the “–single_best_only” option. To construct the annotation file against the assembled genome, the “cdna_alignment_orf_to_genome_orf.pl” command implemented in TransDecoder (Haas BJ, https://github.com/TransDecoder/TransDecoder) was used. The two sets of gene models were merged and provided to the MAKER annotation pipeline (Cantarel *et al.* 2008). Gene models of tomato pre-trained in AUGUSTUS (Stanke *et al.* 2004) were also included. Transposable elements (TEs) were identified using EDTA 2.0 (Ou *et al.* 2019). To filter the gene-related sequences during the detection of TEs, the coding sequences (CDS) were provided. The distribution of genes and TEs was visualized as a circular heatmap generated by Circos (Krzywinski *et al.* 2009).

### Assessment of assembly and annotation quality

The contig number and length distribution of the initial assembly, the assembly after Optical mapping, and the final assembly were obtained by the “stat” command of Seqkit 0.15.0 (Shen *et al.* 2016). L50 statistics and gap content were calculated by the “fx2tab” command of Seqkit with “-nlH -B N” options. The assembly and annotation completeness were assessed by Benchmarking Universal Single-Copy Orthologs (BUSCO; Simão *et al.* 2015) against the genomic sequences and annotated protein sequences available in the Solanales odb10 database (Kriventseva *et al.* 2019).

### Comparison of genome structures

The genome structure of *S. bulbocastanum* was compared with those of six chromosome-scale assemblies: *S. lycopersicum* L. (cv. Heinz 1706 SL5.0; Zhou *et al.* 2022), *S. etuberosum* Lindl. (PG0019 [PI 558302]; Tang *et al.* 2022), *S. verrucosum* (11H23 [PI 666968]; Hosaka *et al.* 2022), *S. chacoense* Bitter (M6 v5; Leisner *et al.* 2018, http://spuddb.uga.edu/M6_v5_0_download.shtml), *S. multidissectum* Hawkes [PG5068, (PI 458379, described as *S. candolleanum* Berthault in Spooner *et al.* 2014); Tang *et al.* 2022], and *S. phureja* (DM v8.1, Yang *et al.* 2023). Chromosomal sequences of these seven genomes were used for pairwise alignment by Minimap2 (Li 2018) and outputted as SAM and PAF files by defining “-ax asm5 -eqx” and “-x ams5” options, respectively. The SAM files were converted to the bam files using SAMtools 1.61.1 (Li *et al.* 2009). Syntenic regions and structural variations were detected using SyRI (Goel *et al.* 2019) with the “-k -F B” option. The results were visualized by Plotsr 0.5.4 (Goel and Schneeberger 2022). Pairwise ratios of syntenic regions were calculated by dividing the length of syntenic areas by the total chromosome length of each species. The PAF files were used for generating dot-plots by D-GENIES (Cabanettes and Klopp 2018).

### Phylogenetic inference

Detection of orthogroups and phylogenetic inference among tuber-bearing *Solanum* species were assessed using OrthoFinder (Emms and Kelly 2015, 2019). In addition to the seven species used for the genome structure comparison, 21 wild species and seven clones of three cultivated species (Tang *et al.* 2022) and *S. melongena* Wall. (Barchi *et al.* 2021) as an outgroup species were used for the analysis. The longest protein sequences of their annotated gene models, located on the chromosomes or primary contigs, were extracted and used as input datasets for OrthoFinder. The phylogenetic relationship was first inferred by the Species Tree Inference from All Genes (STAG) method (Emms and Kelly 2018) with default parameters. Secondly, a multiple sequence alignment (MSA) procedure was performed with “-M msa” and “-A mafft” options, and the single-copy ortholog groups were detected and aligned using MAFFT (Katoh and Standley 2013). The tree was inferred by FastTree 2 (Price *et al.* 2010) implemented in the Orthofinder pipeline. IQ-TREE 2 (Minh *et al.* 2020) was also used for maximum likelihood inference of phylogenetic relationships using “-m MFP” and “-bb 1000” options to automatically calculate the best-fit amino-acid substitution model and to replicate 1,000 times for an ultrafast bootstrap approximation.

### Characterization of unanchored contigs

Unanchored contigs were aligned with each other and with the assembled genome using Minimap2 with the “-x asm5 -P” option. HiFi reads were aligned to the assembled genome using Minimap2 with the “-ax map-hifi” option. Together with genes and TEs, the coverages of unanchored contigs and HiFi reads were visualized in a circular heatmap generated by Circos. TEs in the regions where unanchored contigs were densely aligned were counted by BEDTools 2.30.0 (Quinlan and Hall 2010) with the intersect command. These regions were aligned to the contigs of 23 wild species and six clones of three cultivated species (Tang *et al.* 2022) and to the seven chromosome-scale assemblies using Minimap2 with the “-ax map-hifi” option. The regions with >80% homology were counted.

## Results and discussion

### Genome assembly

We obtained 72.7 Gb (90× coverage, assuming 800 Mb/genome) of HiFi reads using a PacBio Sequel IIe system with an N50 read size of 20.9 kb and an average read size of 21.1 kb. The initial Hifiasm assembly generated 1,586 contigs (N50 = 50.5 Mb) (Table 1). Chromosomes 2, 3, and 5–11 were already composed of single contigs. To improve contig contiguity, we performed Optical mapping. Of the 200.0 Gb (N50 = 345.9 kb) data generated, 155.0 Gb (N50 = 340.1 kb) were filtered and used for de novo assembly, resulting in 94 optical maps with a total length of 638.3 Mb (N50 = 23.3 Mb). By aligning these optical maps to the primary contigs, eight conflicts in the optical maps and 13 disputes in the primary contigs were found and corrected. The optical mapping might be ineffective since the primary contigs were highly contiguous. As a result, 22 hybrid scaffolds with a total length of 653.1 Mb (N50 = 50.5 Mb) were generated. These hybrid scaffolds and 1,577 unanchored contigs totaling 84.7 Mb were error-corrected and scaffolded with Omni-C read pairs using Juicer and a 3D-DNA pipeline (Supplementary Fig. 1, Table 1). Chromosome identities and directions were determined based on the reference sequences of DM v8.1. The final sequence assembly consisted of 737.9 Mb, among which 647.0 Mb were anchored to the 12 chromosomes; 527 and 17 contigs (28.8 and 1.0 Mb, respectively) were those of chloroplast and mitochondrial genomes. The Hifiasm assembly forms many small contigs for nonlinear genomes, such as chloroplast and mitochondrial genomes (Hosaka *et al.* 2022; Sharma *et al.* 2022; Sun *et al.* 2022). The remaining 1,083 contigs (61.0 Mb) were unanchored (Supplementary Table 1).

**Table 1.** Assembly statistics.

	Primary contigs with PacBio reads	Merged contigs after Optical mapping	Final contigs after Hi-C sequencing
Number of contigs	1,586	1,599	1,639
Total size, bp	737,832,402	737,833,609	737,908,709
Longest size, bp	61,881,511	61,881,511	72,187,831
Mean size, bp	465,215.9	461,434.4	450,218.9
N50 size, bp	50,507,639	44,207,125	52,534,229
L50, number	7	7	7
Gap (%)	-	0.000	0.010

### Genome annotation

**Fig. 1.**
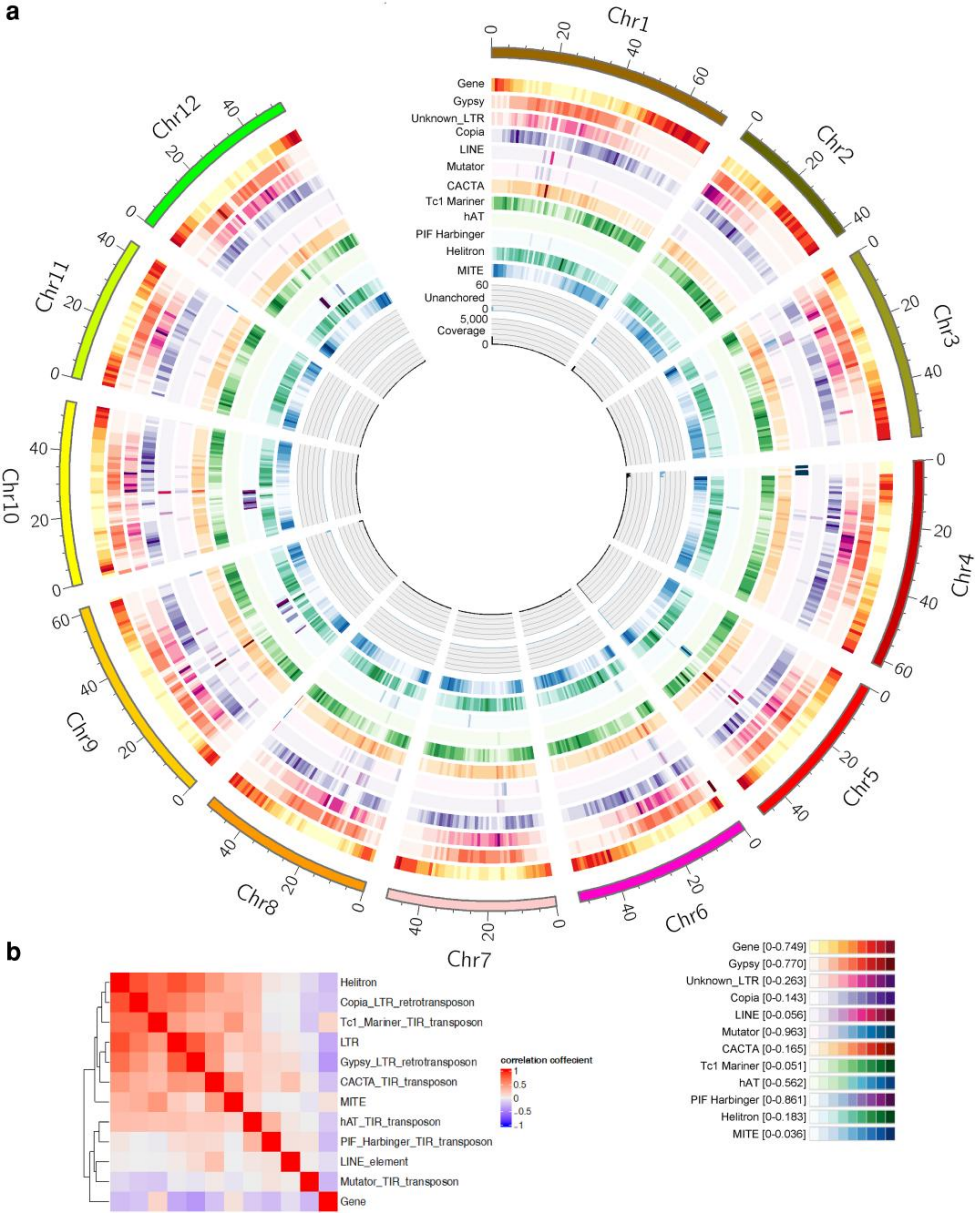
Chromosomal distribution of HiFi reads, unanchored contigs, genes, and transposons (a) and the correlations of their locations (b).

Based on homology, 41,251 out of 56,032 gene models of *S. bulbocastanum* PG6241 were lifted using LiftOff. Using publicly available RNA-seq datasets of *S. bulbocastanum* PG6241, 22,832 protein-coding genes were predicted by TransDecoder. Both gene models and an ab initio pre-trained gene prediction model were used for final annotation by MAKER, and 35,523 genes were predicted. The number of predicted genes was slightly lower than that of DM v8.1 (40,155 genes). TEs were identified by EDTA using CDS of the predicted genes to filter gene-related sequences. As shown in Supplementary Table 2, TEs comprised 56.2% of the genome, which was lower than those of the pangenome of *Solanum* section *Petota* (75.5%; Bozan *et al.* 2023) and *S. phureja* DM v8.1 (60.3%; Yang *et al.* 2023). A significantly higher percentage of TEs was reported for the genome with in vitro propagation in its history (Bozan *et al.* 2023). The presently used *S. bulbocastanum* and previously used *S. verrucosum* clones have been maintained in vitro for almost half a century (Sanetomo and Hosaka 2021). Nevertheless, the *S. bulbocastanum* genome (56.2%) had a lower TE content than the *S. verrucosum* genome (61.8%; Hosaka *et al.* 2022). Among TEs, long terminal repeat (LTR)-type retrotransposons accounted for 26.4%, and terminal inverted repeat (TIR)-type transposons accounted for 18.2%, in which Gypsy (16.5%) and Mutator (8.8%) families were the most abundant, as previously reported in other *Solanum* species (Aversano *et al.* 2015; Gaiero *et al.* 2019; Hosmani *et al.* 2019; Bozan *et al.* 2023). However, the content of Gypsy was much lower in *S. bulbocastanum* than in *S. verrucosum* (24.2%), which is consistent with a finding that the Gypsy content in Mexican diploid species is lower than that in the A-genome species (Gaiero *et al.* 2019; Bozan *et al.* 2023). The genes were densely distributed in subtelomeric regions (Fig. 1a). Some class II transposons, such as Tc1_Mariner and Miniature Inverted-repeat Transposable Elements (MITEs), were distributed in a pattern similar to that of genes (Fig. 1b). In contrast, Gypsy and unknown LTR retrotransposons were densely distributed in pericentromeric regions. Similar distribution patterns have been reported in other species (Zavallo *et al.* 2020; Hosaka *et al.* 2022) except for hAT and PIF Harbinger, which were evenly distributed in the *S. verrucosum* chromosomes (Hosaka *et al.* 2022) but were densely distributed to specific regions in a few chromosomes of *S. bulbocastanum* (Fig. 1a).

### Completeness of genome assembly and annotation

We calculated the LTR Assembly Index (LAI) scores to evaluate the completeness of the genome assembly. The LAI score of the present *S. bulbocastanum* genome was 9.63, slightly higher than that of the *S. bulbocastanum* PG6241 genome (LAI = 8.97). We also assessed the BUSCO scores of genome sequences in eight *Solanum* species, which were reported as either chromosome-scale assemblies or highly contiguous contigs (Supplementary Table 3). In a genome mode, 98.27% of BUSCO genes in Solanales were detected in the present *S. bulbocastanum* genome. The score was comparable to the other species, demonstrating the highly contiguous assembly. However, the BUSCO score of protein sequences was 91.76%, slightly lower than that of PG6241 protein sequences (95.04%). Since we used the RNA-seq datasets of PG6241, some genes were missed during annotation, possibly due to the sequence variations between the *S. bulbocastanum* strains (Supplementary Table 3).

### Synteny and structural variation among *Solanum* species

**Fig. 2.**
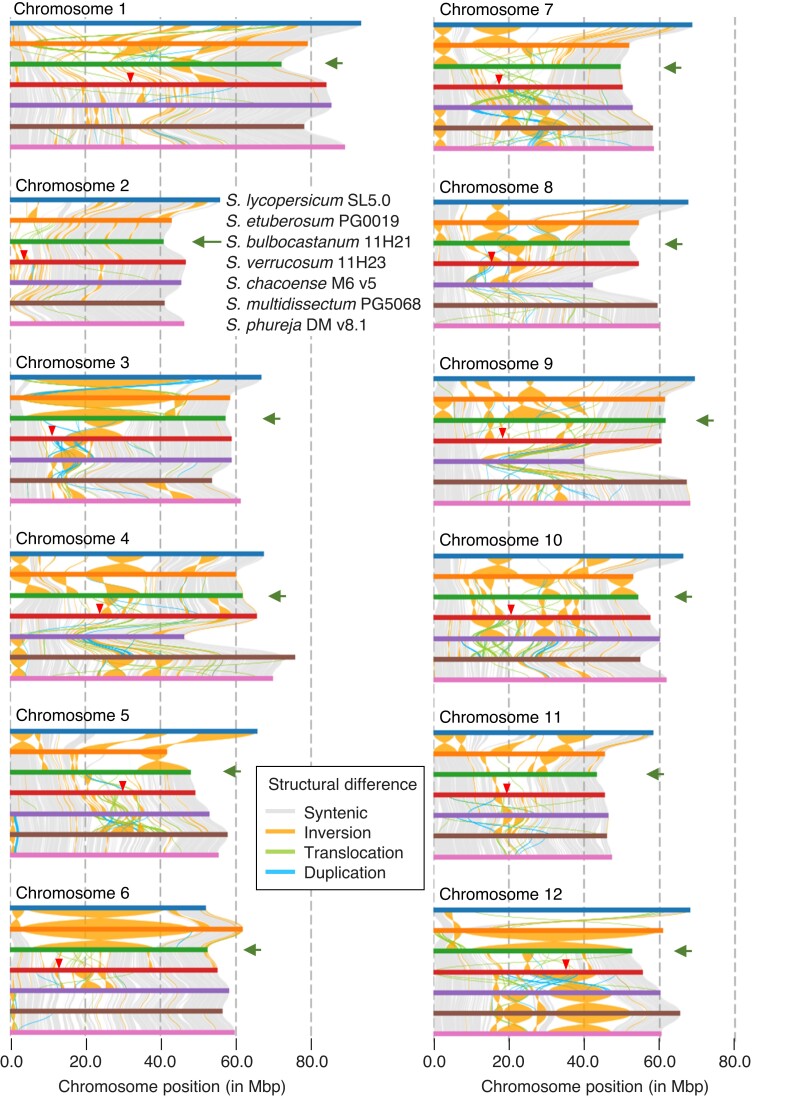
Length and structural variation among seven chromosome-scale assemblies. Arrowed chromosomes are those of *S. bulbocastanum*. Arrowheads indicate the locations of putative centromeres in the *S. verrucosum* genome (Hosaka *et al.* 2022).

**Fig. 3.**
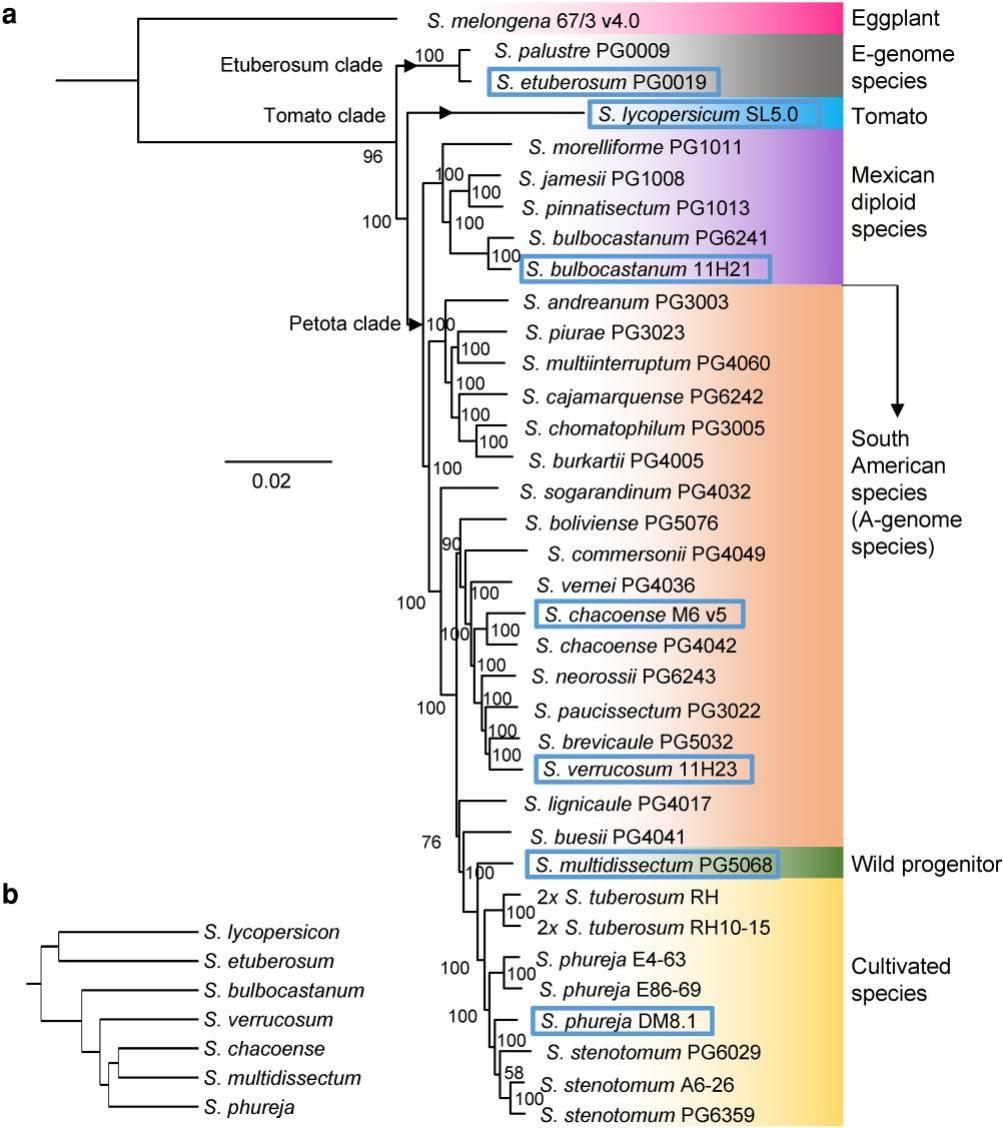
Species relationships based on genome structures (a) and gene sequence similarities (b). a) A dendrogram, constructed using an UPGMA, based on pairwise ratios of syntenic regions among seven chromosome-scale assemblies, showing genome structure similarity. b) A phylogenetic tree generated using IQ-TREE 2, constructed using single-copy gene sequence similarities in 1,624 orthogroups commonly present in all 36 species. A bootstrap value is given in each node. Species with chromosome-scale assemblies are shown in rectangles. The distinctiveness of *S. bulbocastanum* and other Mexican diploid species from the A-genome species is disclosed. Note that the species names used are those described by Hawkes (1990). According to Spooner's taxonomy, *S.*  *multidissectum* is included in a superspecies *S. candolleanum*, while *S. stenotomum* and *S. phureja* are included in *S. tuberosum* (Spooner *et al.* 2014).

Structural differences among seven chromosome-scale assemblies were compared. The chromosomal length of *S. bulbocastanum* was the shortest: 7.4% shorter than that of *S. phureja* DM v 8.1 (Supplementary Table 4 and Fig. 2). This is consistent with the finding that the genomes of Mexican diploid species are smaller in size and contained fewer TEs than those of A-genome species (Bozan *et al.* 2023). Dot-plots using D-Genies disclosed low sequence similarities and frequent losses of linearity in pericentromeric and centromere regions (Supplementary Figs. 2–4). Syntenic regions were further identified by SyRI (Fig. 2). Subtelomeric regions of chromosomes tended to be more conserved than pericentromeric regions, as previously described (Hosaka *et al.* 2022; Tang *et al.* 2022). Small but frequent pericentromeric inversions between the *S. bulbocastanum* and *S. verrucosum* genomes were found in chromosomes 1–4, 8–10, and 12, which might coincide with observations of minor structural chromosome differences at pachytene in the F_1_ of *S. verrucosum* × *S. bulbocastanum* (Hermsen and Ramanna 1976). Furthermore, in addition to frequent small inversions, large centromeric inversions between the *S. bulbocastanum* and *S. etuberosum* genomes were observed in chromosomes 3, 6, 8, 9, and 12, which likely caused irregular meiosis as observed in F_1_ hybrids between Mexican diploid species and *Etuberosa* series species (Ramanna and Hermsen 1981). To represent structural differences, pairwise ratios of syntenic regions were calculated among seven chromosome-scale assemblies (Supplementary Table 5) and visualized as a dendrogram constructed by an unweighted pair group method with arithmetic mean (UPGMA) (Fig. 3a). *S. chacoense* and *S. multidissectum* were clustered first, then with *S. phureja* and *S. verrucosum*, resulting in an A-genome species group. Since *S. multidissectum* is one of the putative ancestral species of cultivated potatoes, as shown in Fig. 3b (Spooner *et al.* 2005; Sukhotu and Hosaka 2006), there might be a possibility of misassembly in either the *S. chacoense* or *S. multidissectum* genomes. Highly repetitive sequences around a centromere might cause a large misassembled region (a centromeric inversion), as occurred in chromosome 12 of the *S. phureja* genome (DM v4.04), which was inverted in DM v6.1 (Pham *et al.* 2020). Consequently, this group was clustered with *S. bulbocastanum*, forming a tuber-bearing species group (=section *Petota* or Petota clade). The tuber-bearing species group was then clustered with a group of *S. etuberosum* (Etuberosum clade) and tomato (Tomato clade).

### Phylogenetic inference

OrthoFinder assigned 1,289,183 genes identified in 36 *Solanum* species and clones into 36,033 orthogroups (Supplementary Table 6). Among the 36,033 orthogroups, 5,905 (16.4%) were present in all the species and clones analyzed. These were used to infer the species’ phylogeny using OrthoFinder with the STAG algorithm (Supplementary Fig. 5a). Of the 5,905 common orthogroups, 1,624 were single-copy orthogroups, which were used to infer the species’ phylogeny using the MSA method with FastTree 2 and IQ-TREE 2 (Supplementary Fig. 5b and Fig. 3b, respectively). As expected, three phylogenetic trees indicated the present *S. bulbocastanum* located at the same node of *S. bulbocastanum* PG6241, showing that the two genomes are sister genomes and, together with other Mexican diploid species, formed a distinct cluster from the A-genome species, most of which are distributed in South America and created a large group including cultivated species and its wild ancestor (Fig. 3b). Noteworthy, *S. verrucosum*, only A-genome species distributed in Mexico, was located in the cluster of A-genome species. The presence of *S. verrucosum* in Mexico supports the suggestion that an A-genome species dispersed to Mexico from South America (Hawkes and Jackson 1992). The phylogenetic tree in Fig. 3b was similar to that based on sequence similarity in 3,971 single-copy genes, both using the same software IQ-TREE 2 (Tang *et al.* 2022). The two phylogenetic trees suggested that E-genome species share a most recent common ancestor with the clade comprising tomato and tuber-bearing species. However, the phylogenetic trees generated by STAG and FastTree 2 suggest that the Tomato clade is a sister to a clade comprising E-genome and tuber-bearing species’ shared ancestor (Supplementary Fig. 5a, b). Both branching patterns (the Etuberosum clade sister to the Tomato-Petota clade or the Tomato clade sister to the Etuberosum-Petota clade) have previously been reported and argued (Hosaka *et al.* 1984; Spooner *et al.* 1993; Huang *et al.* 2019; Gagnon *et al.* 2022; Tang *et al.* 2022). Structural differences among these genomes exhibited a slightly different branching pattern as described earlier (Fig. 3a). Tang *et al.* (2022) mentioned that a homologous gene to *Identity of Tuber 1*, an essential gene for initiating potato tubers, was identified but unfunctional in E-genome species. The TE profiles support the idea that *S. etuberosum* is more similar to the potato than tomato species (Gaiero *et al.* 2019). Further research is needed to solve this discordance using multiple species and accessions of the E-genome species.

### Characterization of the unanchored contigs

**Fig. 4.**
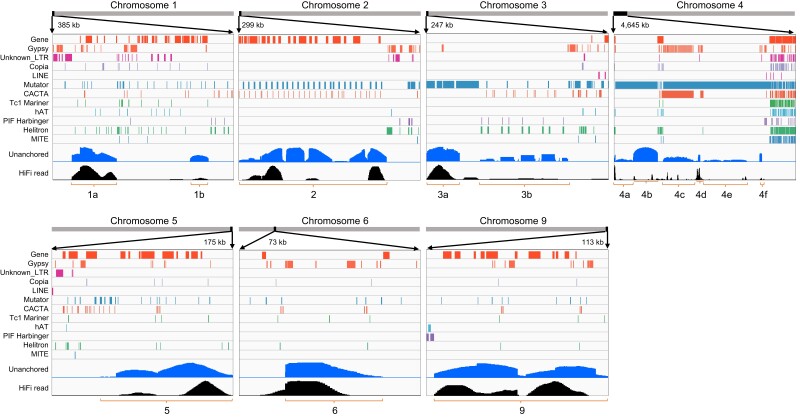
Distribution of genes, transposons, and dosages of HiFi reads and unanchored contigs in the unanchored-contig clustered regions (UCCRs 1a to 9).

Since 1,083 contigs remained unanchored, we further characterized these unanchored contigs. A pairwise comparison of these contigs by Minimap2 (Supplementary Fig. 6a) indicated their highly repetitive nature and shared similarities. Most of these contigs were aligned to the telomeric regions of chromosomes 1–5, and 9, and the subtelomeric region of chromosome 6 (Supplementary Fig. 6b). Interestingly, in these regions, the coverages of HiFi reads were abnormally high, suggesting that their highly repetitive nature made it difficult to assemble even with long reads and caused underestimation of repeat numbers and many unanchored contigs. Based on the coverages of the unanchored contigs and HiFi reads, 14 regions (1a–9) were tentatively identified as unanchored-contig clustered regions (UCCRs) with a size range from 39.7 kb (1b) to 1.14 Mb (4e) (Fig. 4, Supplementary Table 7). The UCCRs were composed of various repeat sequences and arrays of specific TEs: Gypsy (1a, 1b, 4c, 5, 6, and 9), helitron (3b), CACTA (2 and 4c), or Mutator (2, 3a, 4a, 4b, 4c, 4d, 4e, and 4f) (Supplementary Table 7). The UCCRs were aligned using Minimap2 with >80% homology to the contigs constructed from 23 wild species and 6 clones of three cultivated species (Tang *et al.* 2022). The UCCRs 1b and 6 were found in all species, while 1a and 5 were found in 19 and 11 among 29 species and clones, respectively (Supplementary Table 8). The other UCCRs were rare. In *S. bulbocastanum* PG6241, only five (1b, 3a, 4b, 4e, and 6) were found, but the most significant number of UCCRs was found. The UCCR sequences were aligned with >80% homology to seven chromosome-scale assemblies (Supplementary Table 9). Except for the present *S. bulbocastanum* genome, only a few had the same UCCRs: the UCCR 2 in *S. multidissectum* and 1a, 1b, 6, and 9 in *S. lycopersicum*. Therefore, not all but most of these highly repetitive sequences, or UCCRs, are very specific to the present *S. bulbocastanum* genome. Although a further survey is needed, these UCCRs likely originated by in vitro propagation that continued over half a century, as Bozan *et al.* (2023) suggested.

## Conclusions

A de novo chromosome-scale assembly of the *S. bulbocastanum* genome was constructed, which was slightly shorter and had less content of TEs compared with the A-genome. It was distinct from the A-genome in a syntenic relationship and sequence homology of orthologous genes. As noted in our and other studies, it is most closely related to the Mexican diploid species, excluding *S. verrucosum* (Hosaka *et al.* 1984; Spooner *et al.* 1991, 2008; Pendinen *et al.* 2008; Rodríguez and Spooner 2009; Li *et al.* 2018; Huang *et al.* 2019; Tang *et al.* 2022; Bozan *et al.* 2023). These findings provide important insights into understanding the genome evolution of wild potato species and the genetic diversity of Mexican diploid species. Due to reproductive barriers, the use of Mexican diploid species in potato breeding has been mainly limited to that for late blight resistance (Song *et al.* 2003; Lokossou *et al.* 2010; Sanetomo *et al.* 2019). Whole-genome sequences of *S. bulbocastanum* could help to understand and break a genetic mechanism for reproductive barriers, which facilitates the incorporation of valuable traits such as resistance to various viruses, aphids, Colorado potato beetle, and nematodes, and heat tolerance (Hawkes 1958, 1990; Hanneman 1989) into breeder's gene pools.

## Supplementary Material

jkae080_Supplementary_Data

## Data Availability

The raw DNA sequencing reads, genome assembly, and annotation have been deposited into the National Center for Biotechnology Information under BioProject Number PRJNA1009588. Supplemental material is available at G3 online. Supplemental material available at G3 online.
