# Supplementary material for: A de novo genome assembly of Solanum bulbocastanum Dun., a Mexican diploid species reproductively isolated from the A-genome species, including cultivated potatoes
Source: G3 (Bethesda). 2024 Apr 12;14(6):jkae080. doi: 10.1093/g3journal/jkae080 (PMC11152074; doi:10.1093/g3journal/jkae080)
Supplement: jkae080_Supplementary_Data [file jkae080_supplementary_data.zip › Supplementary Figure 3.pptx]

## Slide 1
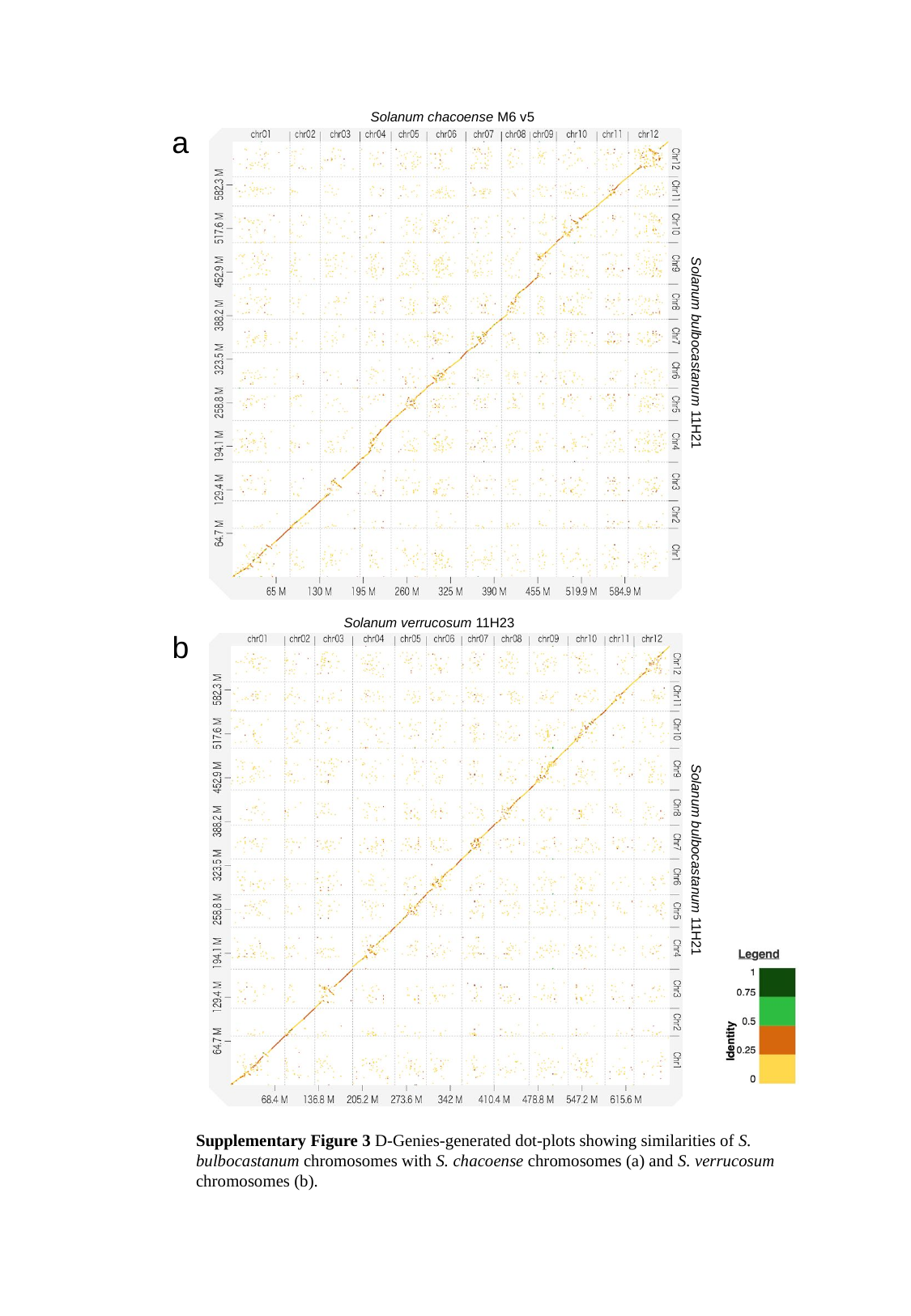

Solanum chacoense M6 v5
a
Solanum bulbocastanum 11H21
Solanum verrucosum 11H23
b
Solanum bulbocastanum 11H21
Supplementary Figure 3 D-Genies-generated dot-plots showing similarities of S. bulbocastanum chromosomes with S. chacoense chromosomes (a) and S. verrucosum chromosomes (b).
