# Supplementary material for: A de novo genome assembly of Solanum bulbocastanum Dun., a Mexican diploid species reproductively isolated from the A-genome species, including cultivated potatoes
Source: G3 (Bethesda). 2024 Apr 12;14(6):jkae080. doi: 10.1093/g3journal/jkae080 (PMC11152074; doi:10.1093/g3journal/jkae080)
Supplement: jkae080_Supplementary_Data [file jkae080_supplementary_data.zip › Supplementary Figure 4.pptx]

## Slide 1
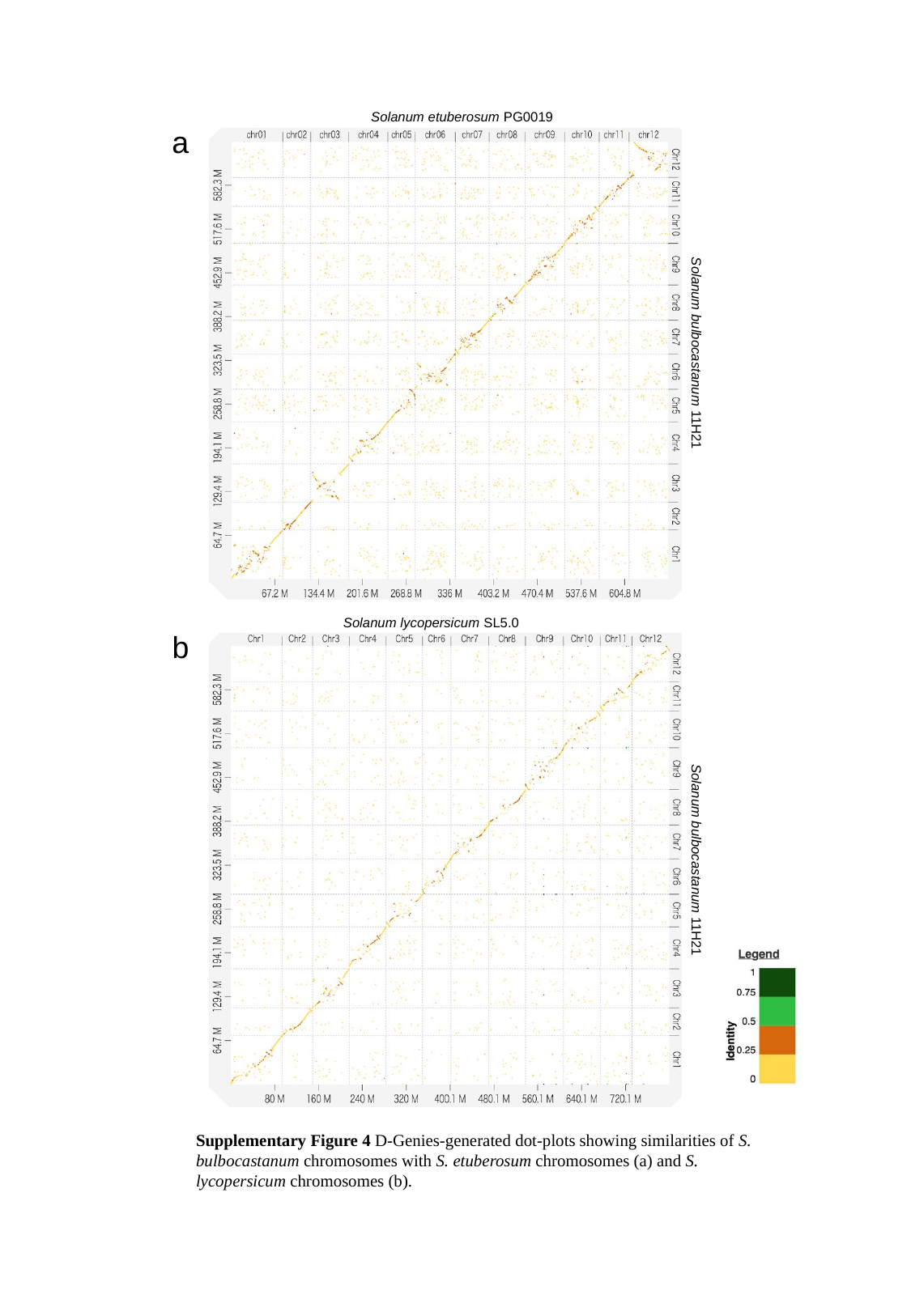

Solanum etuberosum PG0019
a
Solanum bulbocastanum 11H21
Solanum lycopersicum SL5.0
b
Solanum bulbocastanum 11H21
Supplementary Figure 4 D-Genies-generated dot-plots showing similarities of S. bulbocastanum chromosomes with S. etuberosum chromosomes (a) and S. lycopersicum chromosomes (b).
