# Supplementary material for: A de novo genome assembly of Solanum bulbocastanum Dun., a Mexican diploid species reproductively isolated from the A-genome species, including cultivated potatoes
Source: G3 (Bethesda). 2024 Apr 12;14(6):jkae080. doi: 10.1093/g3journal/jkae080 (PMC11152074; doi:10.1093/g3journal/jkae080)
Supplement: jkae080_Supplementary_Data [file jkae080_supplementary_data.zip › Supplementary Figure 5.pptx]

## Slide 1
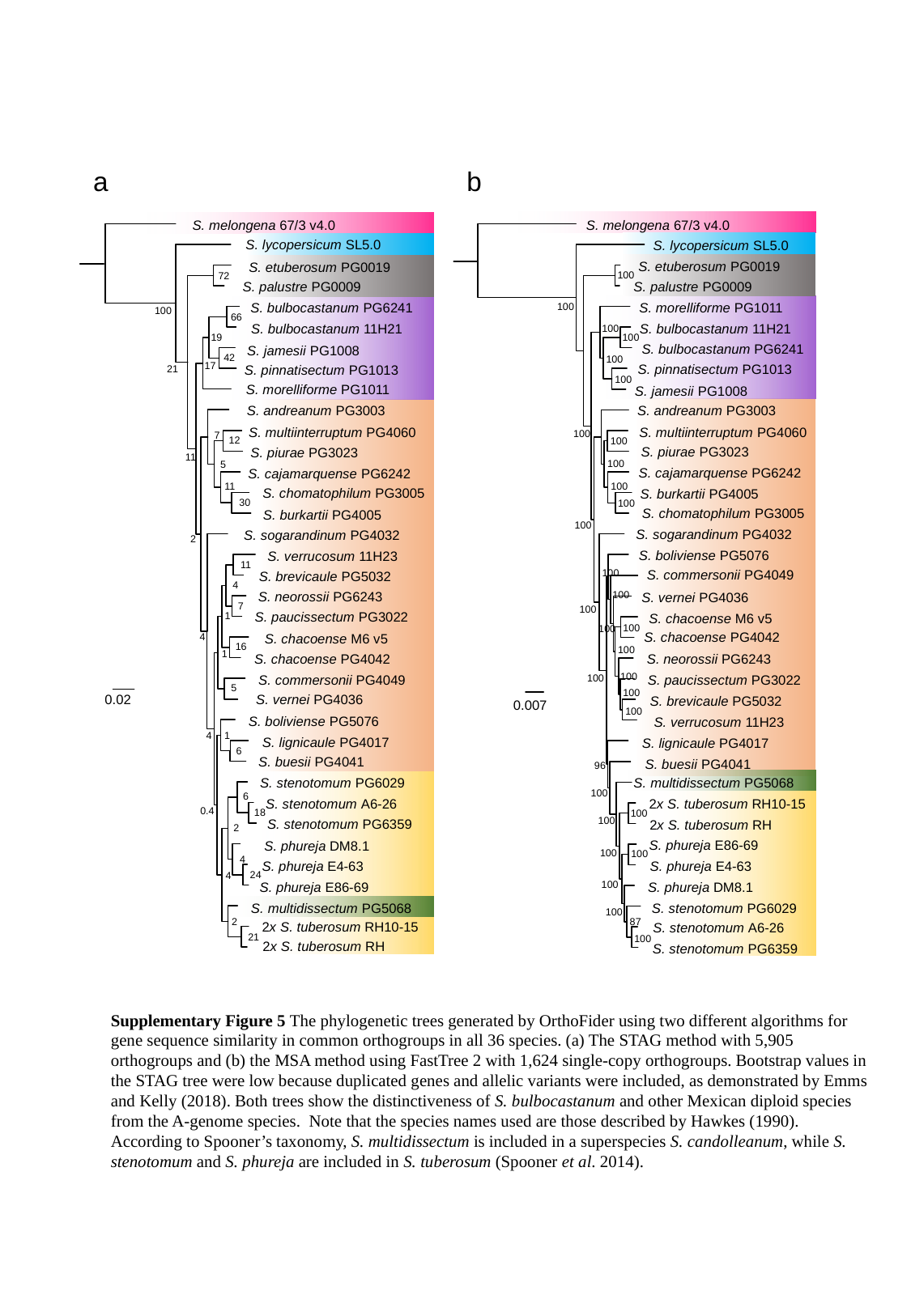

a
b
S. melongena 67/3 v4.0
S. lycopersicum SL5.0
S. etuberosum PG0019
100
S. palustre PG0009
S. morelliforme PG1011
100
S. bulbocastanum 11H21
100
100
S. bulbocastanum PG6241
100
S. pinnatisectum PG1013
100
S. jamesii PG1008
S. andreanum PG3003
S. multiinterruptum PG4060
100
100
S. piurae PG3023
100
S. cajamarquense PG6242
100
S. burkartii PG4005
100
S. chomatophilum PG3005
100
S. sogarandinum PG4032
S. boliviense PG5076
100
S. commersonii PG4049
100
S. vernei PG4036
100
S. chacoense M6 v5
100
100
S. chacoense PG4042
100
S. neorossii PG6243
100
S. paucissectum PG3022
100
100
S. brevicaule PG5032
0.007
100
S. verrucosum 11H23
S. lignicaule PG4017
S. buesii PG4041
96
S. multidissectum PG5068
100
2x S. tuberosum RH10-15
100
100
2x S. tuberosum RH
S. phureja E86-69
100
100
S. phureja E4-63
100
S. phureja DM8.1
S. stenotomum PG6029
100
87
S. stenotomum A6-26
100
S. stenotomum PG6359
S. melongena 67/3 v4.0
S. lycopersicum SL5.0
S. etuberosum PG0019
72
S. palustre PG0009
S. bulbocastanum PG6241
100
66
S. bulbocastanum 11H21
19
S. jamesii PG1008
42
17
S. pinnatisectum PG1013
21
S. morelliforme PG1011
S. andreanum PG3003
S. multiinterruptum PG4060
7
12
S. piurae PG3023
11
5
S. cajamarquense PG6242
11
S. chomatophilum PG3005
30
S. burkartii PG4005
S. sogarandinum PG4032
2
S. verrucosum 11H23
11
S. brevicaule PG5032
4
S. neorossii PG6243
7
S. paucissectum PG3022
1
S. chacoense M6 v5
4
16
1
S. chacoense PG4042
S. commersonii PG4049
5
0.02
S. vernei PG4036
S. boliviense PG5076
4
1
S. lignicaule PG4017
6
S. buesii PG4041
S. stenotomum PG6029
6
S. stenotomum A6-26
0.4
18
S. stenotomum PG6359
2
S. phureja DM8.1
4
S. phureja E4-63
24
4
S. phureja E86-69
S. multidissectum PG5068
2
2x S. tuberosum RH10-15
21
2x S. tuberosum RH
Supplementary Figure 5 The phylogenetic trees generated by OrthoFider using two different algorithms for gene sequence similarity in common orthogroups in all 36 species. (a) The STAG method with 5,905 orthogroups and (b) the MSA method using FastTree 2 with 1,624 single-copy orthogroups. Bootstrap values in the STAG tree were low because duplicated genes and allelic variants were included, as demonstrated by Emms and Kelly (2018). Both trees show the distinctiveness of S. bulbocastanum and other Mexican diploid species from the A-genome species. Note that the species names used are those described by Hawkes (1990). According to Spooner’s taxonomy, S. multidissectum is included in a superspecies S. candolleanum, while S. stenotomum and S. phureja are included in S. tuberosum (Spooner et al. 2014).
