# Supplementary material for: A de novo genome assembly of Solanum bulbocastanum Dun., a Mexican diploid species reproductively isolated from the A-genome species, including cultivated potatoes
Source: G3 (Bethesda). 2024 Apr 12;14(6):jkae080. doi: 10.1093/g3journal/jkae080 (PMC11152074; doi:10.1093/g3journal/jkae080)
Supplement: jkae080_Supplementary_Data [file jkae080_supplementary_data.zip › Supplementary Figure 6.pptx]

## Slide 1
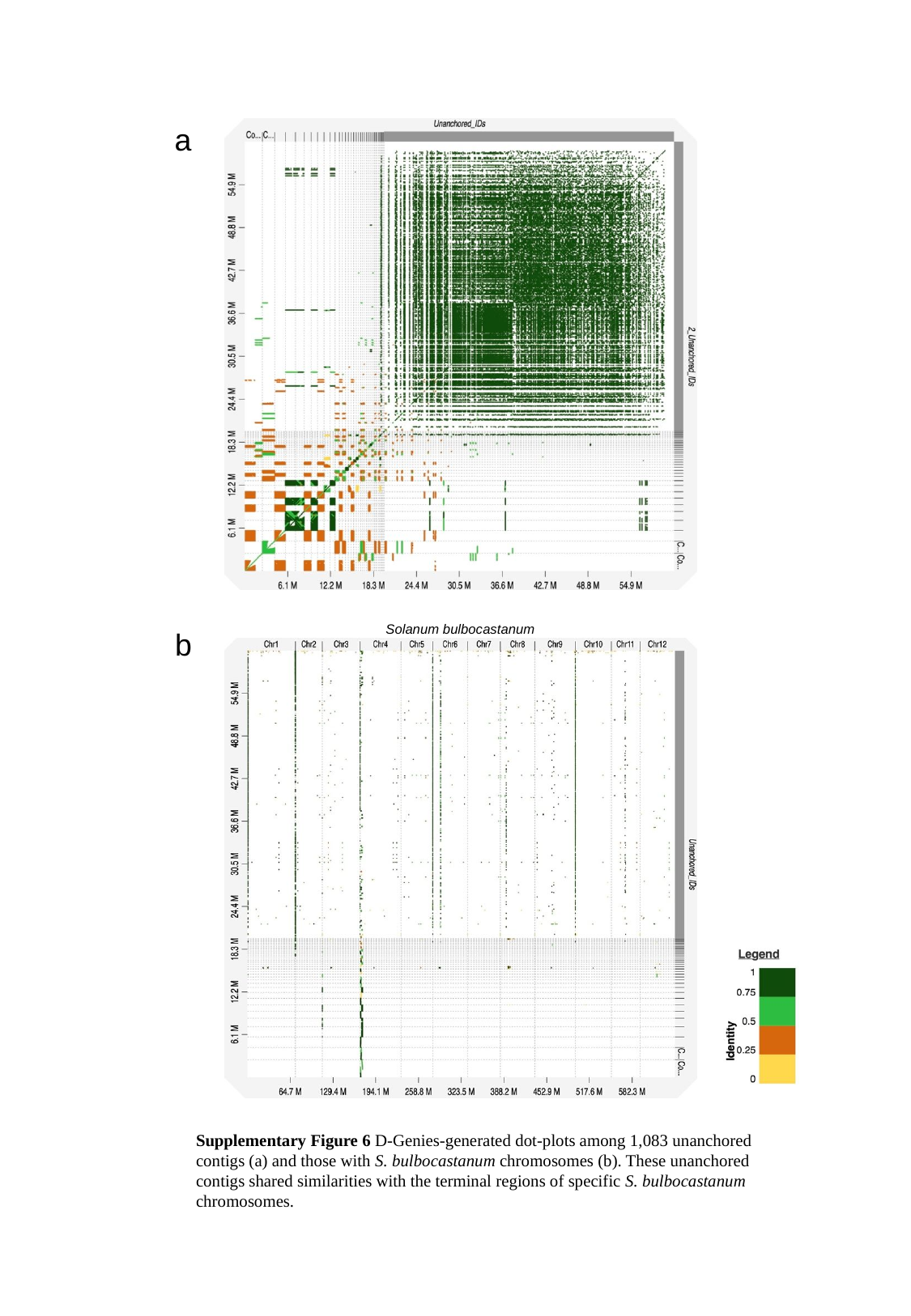

a
Solanum bulbocastanum
b
Supplementary Figure 6 D-Genies-generated dot-plots among 1,083 unanchored contigs (a) and those with S. bulbocastanum chromosomes (b). These unanchored contigs shared similarities with the terminal regions of specific S. bulbocastanum chromosomes.
