# Supplementary material for: A de novo genome assembly of Solanum bulbocastanum Dun., a Mexican diploid species reproductively isolated from the A-genome species, including cultivated potatoes
Source: G3 (Bethesda). 2024 Apr 12;14(6):jkae080. doi: 10.1093/g3journal/jkae080 (PMC11152074; doi:10.1093/g3journal/jkae080)
Supplement: jkae080_Supplementary_Data [file jkae080_supplementary_data.zip › Supplementary_Figure_1_G3-2024-404889.pptx]

## Slide 1
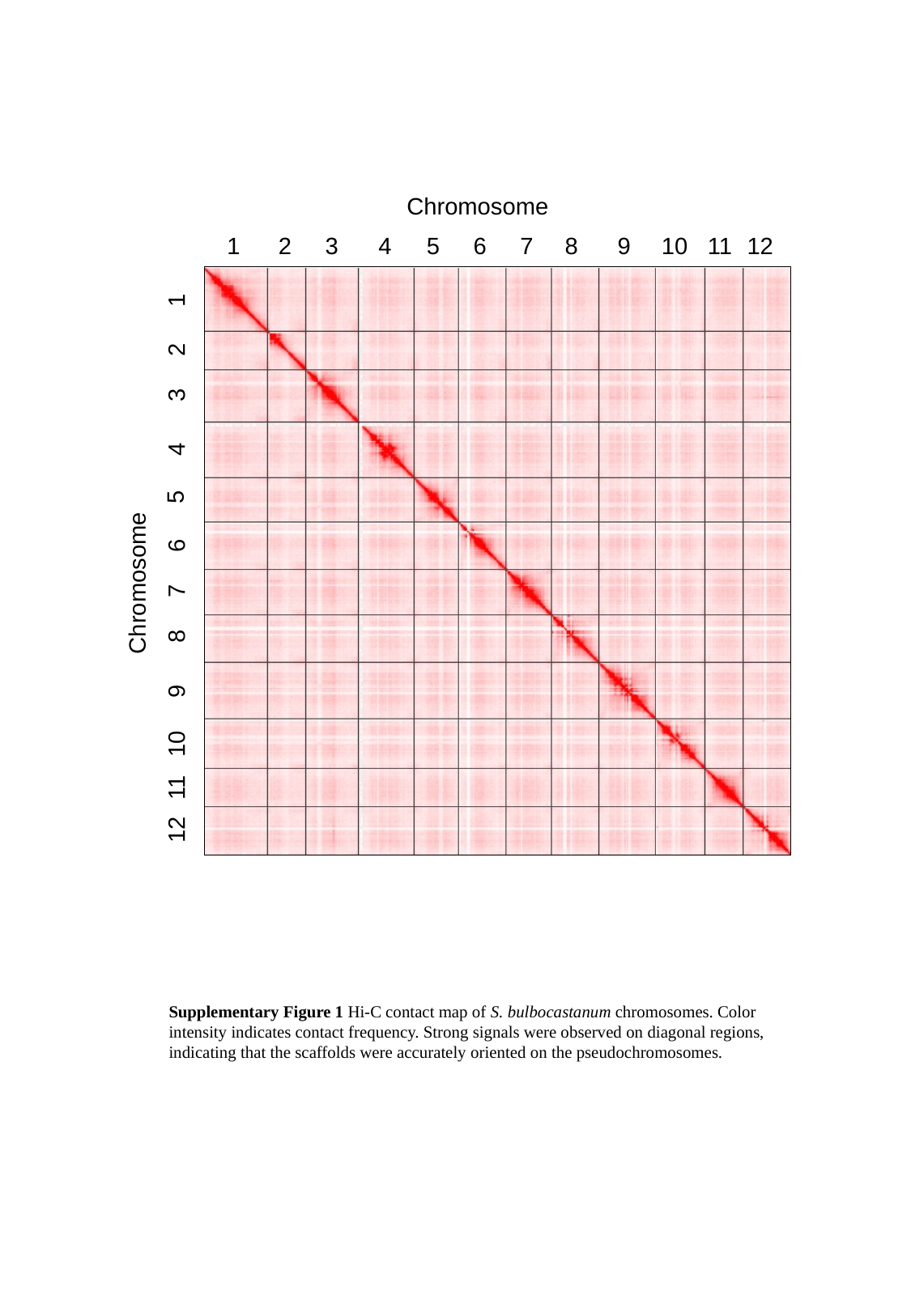

Chromosome
1
2
3
4
5
6
7
8
9
10
11
12
1
2
3
4
5
6
Chromosome
7
8
9
10
11
12
Supplementary Figure 1 Hi-C contact map of S. bulbocastanum chromosomes. Color intensity indicates contact frequency. Strong signals were observed on diagonal regions, indicating that the scaffolds were accurately oriented on the pseudochromosomes.
