# Supplementary material for: A de novo genome assembly of Solanum bulbocastanum Dun., a Mexican diploid species reproductively isolated from the A-genome species, including cultivated potatoes
Source: G3 (Bethesda). 2024 Apr 12;14(6):jkae080. doi: 10.1093/g3journal/jkae080 (PMC11152074; doi:10.1093/g3journal/jkae080)
Supplement: jkae080_Supplementary_Data [file jkae080_supplementary_data.zip › Supplementary_Tables_G3-2024-404889.docx]

**Supplementary Table 1.** Final contigs after Hi-C sequencing composing the *S. bulbocastanum* genome.

| Category | Number | Total length (bp) | Mean length (bp) | N50 (bp) |
| --- | --- | --- | --- | --- |
| Chromosome | 12 | 647,003,022 | 53,916,918.5 | 52,733,367 |
| Chloroplast | 527 | 28,831,008 | 54,707.8 | 52,443 |
| Mitochondria | 17 | 1,047,513 | 61,618.4 | 68,761 |
| Unanchored | 1,083 | 61,027,166 | 56,350.1 | 51,959 |
| Total | 1,639 | 737,908,709 | 450,218.9 | 52,534,229 |

**Supplementary Table 2.** Transposable element (TE) density in the *S. bulbocastanum* genome.

| Type | TE | Number^1)^ | Total size (bp) | Ratio (%) |
| --- | --- | --- | --- | --- |
| LTR | Copia | 52755 | 31683347 | 4.29% |
|  | Gypsy | 139088 | 121503833 | 16.47% |
|  | unknown | 68729 | 41936044 | 5.68% |
| TIR | CACTA | 59710 (3732) | 20689466 | 2.80% |
|  | Mutator | 111885 (12184) | 64746485 | 8.78% |
|  | PIF Harbinger | 28205 (1570) | 22417797 | 3.04% |
|  | Tc1 Mariner | 50176 (10691) | 12271412 | 1.66% |
|  | hAT | 32844 (9990) | 14042275 | 1.90% |
| non-LTR | LINE element | 2341 | 1363942 | 0.18% |
|  | unknown | 5 | 555 | 0.00% |
| non-LTR | helitron | 108646 | 39957278 | 5.42% |
| Repeat region |  | 120208 | 44123530 | 5.98% |
| Total |  | 774592 | 414735964 | 56.21% |

^1)^The number of Miniature Inverted-repeat Transposable Elements (MITEs) in parentheses.

**Supplementary Table 3.** BUSCO statistics of the chromosome-scale assembly of *S. bulbocastanum* compared with other six chromosome-scale assemblies and with highly contiguous contigs of *S. bulbocastanum* PG6241.

| BUSCOs | *S. phureja* | *S. multidissectum* | *S. chacoense* | *S. verrucosum* | *S. bulbocastanum* (this study) | *S. bulbocastanum*  (PG6241) | *S. etuberosum* | *S. lycopersicum* |
| --- | --- | --- | --- | --- | --- | --- | --- | --- |
| Genome mode |  |  |  |  |  |  |  |  |
| Complete | 5861  (98.50%) | 5822  (97.85%) | 5651  (94.97%) | 5854  (98.39%) | 5847  (98.27%) | 5861  (98.50%) | 5847  (98.27%) | 5852  (98.35%) |
| Complete and single-copy | 5724  (96.2%) | 5645  (94.87%) | 5506  (92.54%) | 5701  (95.82%) | 5711  (95.98%) | 2381  (40.02%) | 5715  (96.05%) | 5745  (96.55%) |
| Complete and duplicated | 137  (2.30%) | 177  (2.97%) | 145  (2.44%) | 153  (2.57%) | 136  (2.29%) | 3480  (58.49%) | 132  (2.22%) | 107  (1.80%) |
| Fragmented | 8  (0.13%) | 8  (0.13%) | 11  (0.18%) | 8  (0.13%) | 12  (0.20%) | 8  (0.13%) | 10  (0.17%) | 12  (0.20%) |
| Missing | 81  (1.36%) | 120  (2.02%) | 288  (4.84%) | 88  (1.48%) | 91  (1.53%) | 81  (1.36%) | 93  (1.56%) | 86  (1.45%) |
| Protein mode |  |  |  |  |  |  |  |  |
| Complete | 5791  (97.33%) | 5713  (96.02%) | 5519  (92.76%) | 5489  (92.25%) | 5460  (91.76%) | 5655  (95.04%) | 5570  (93.61%) | 5643  (94.84%) |
| Complete and single-copy | 5626  (94.55%) | 1430  (24.03%) | 2883  (48.45%) | 5318  (89.38%) | 5323  (89.46%) | 2392  (40.20%) | 5277  (88.69%) | 4784  (80.40%) |
| Complete and duplicated | 165  (2.77%) | 4283  (71.98%) | 2636  (44.30%) | 171  (2.87%) | 137  (2.30%) | 3263  (54.84%) | 293  (4.92%) | 859  (14.44%) |
| Fragmented | 37  (0.62%) | 56  (0.94%) | 119  (2.00%) | 188  (3.16%) | 37  (0.62%) | 73  (1.23%) | 53  (0.89%) | 53  (0.89%) |
| Missing | 122  (2.05%) | 181  (3.04%) | 312  (5.24%) | 273  (4.59%) | 453  (7.61%) | 222  (3.73%) | 327  (5.50%) | 254  (4.27%) |

**Supplementary Table 4.** Comparison of total assembly size (bp) among seven chromosome-scale assemblies, revealing the shortest total assembly size (bp) in *S. bulbocastanum*.

| Chromosome | *S. phureja* | *S. multidissectum* | *S. chacoense* | *S. verrucosum* | *S. bulbocastanum* | *S. etuberosum* | *S. lycopersicum* |
| --- | --- | --- | --- | --- | --- | --- | --- |
| 1 | 89006753 | 78219642 | 85473275 | 84109000 | 72187831 | 79206479 | 93364382 |
| 2 | 46410555 | 41156863 | 45628993 | 46727947 | 40936425 | 43066457 | 55809412 |
| 3 | 61344578 | 53673481 | 58955458 | 58981818 | 57313237 | 58488985 | 66889691 |
| 4 | 69908028 | 75809500 | 46305968 | 65655322 | 61934191 | 60087454 | 67567563 |
| 5 | 55421548 | 57921114 | 53122106 | 49370500 | 48117991 | 41822827 | 65743948 |
| 6 | 59778245 | 56496519 | 58321281 | 55157000 | 52534229 | 61842509 | 52165627 |
| 7 | 58565559 | 58303995 | 52927395 | 50238081 | 49779594 | 52049262 | 68659810 |
| 8 | 60103403 | 59479824 | 42309432 | 54563706 | 52107142 | 54508690 | 67617550 |
| 9 | 68138841 | 67292995 | 40015257 | 60548446 | 61628006 | 61493670 | 69406105 |
| 10 | 61958337 | 54888067 | 60108243 | 57571353 | 54371561 | 53029253 | 66347229 |
| 11 | 47505424 | 46061505 | 46538378 | 45502451 | 43359448 | 45543602 | 58414624 |
| 12 | 60495205 | 65536000 | 60228640 | 55551619 | 52733367 | 60896088 | 68130381 |
| Total size | 738636476 | 714839505 | 649934426 | 683977243 | 647003022 | 672035276 | 800116322 |

**Supplementary Table 5.** Pairwise ratios of syntenic regions among seven chromosome-scale assemblies.

| Species | *S. lycopersicum* | *S. etuberosum* | *S. bulbocastanum* | *S. verrucosum* | *S. chacoense* | *S. multidissectum* | *S. phureja* |
| --- | --- | --- | --- | --- | --- | --- | --- |
| *S. lycopersicum* | 1.000 | 0.263 | 0.335 | 0.367 | 0.383 | 0.359 | 0.338 |
| *S. etuberosum* | 0.246 | 1.000 | 0.337 | 0.350 | 0.350 | 0.339 | 0.322 |
| *S. bulbocastanum* | 0.305^1)^ | 0.300 | 1.000 | 0.530 | 0.540 | 0.511 | 0.508 |
| *S. verrucosum* | 0.320 | 0.292 | 0.508 | 1.000 | 0.611 | 0.583 | 0.578 |
| *S. chacoense* | 0.310 | 0.279 | 0.487 | 0.590 | 1.000 | 0.574 | 0.639 |
| *S. multidissectum* | 0.306 | 0.288 | 0.491 | 0.593 | 0.614 | 1.000 | 0.673 |
| *S. phureja* | 0.288 | 0.289 | 0.498 | 0.599 | 0.705 | 0.655 | 1.000 |

The ratio of the syntenic region, for example, was calculated by the length of the syntenic region of the *S. bulbocastanum* genome against the *S. lycopersicum* genome (244,054,584 bp) divided by the total length of the *S. lycopersicum* genome (800,116,322 bp).

**Supplementary Table 6.** OrthoFinder disclosed 5,905 orthogroups in common in 36 species and clones, of which 1,624 were single-copy orthogroups used to infer the species' phylogeny in Fig. 3B.

| Statistics |  |
| --- | --- |
| Number of species | 36 |
| Number of genes | 1,289,183 |
| Number of genes in orthogroups | 1,247,258 |
| Number of unassigned genes | 41,925 |
| Percentage of genes in orthogroups | 96.7 |
| Percentage of unassigned genes | 3.3 |
| Number of orthogroups | 36,033 |
| Number of species-specific orthogroups | 2,302 |
| Number of genes in species-specific orthogroups | 9,828 |
| Percentage of genes in species-specific orthogroups | 0.8 |
| Mean orthogroup size | 34.6 |
| Median orthogroup size | 32.0 |
| G50 (assigned genes) | 48 |
| G50 (all genes) | 45 |
| O50 (assigned genes) | 5,800 |
| O50 (all genes) | 6,253 |
| Number of orthogroups with all species present | 5,905 |
| Number of single-copy orthogroups | 1,624 |

**Supplementary Table 7.** The unanchored-contig clustered regions (UCCRs 1a–9) in the *S. bulbocastanum* genome consisted of TEs and repeat sequences.

| UCCR  identity | Chr | Position | Length (bp) | TE number | Classification | No. |
| --- | --- | --- | --- | --- | --- | --- |
| 1a | 1 | 39,967-136,810 | 96,844 | 00000189 | repeat region | 10 |
|  |  |  |  | 00002782 INT | Gypsy LTR retrotransposon | 7 |
|  |  |  |  | 00001454 INT | Gypsy LTR retrotransposon | 6 |
|  |  |  |  | 00000017 | repeat region | 3 |
|  |  |  |  | 00000027 | repeat region | 3 |
|  |  |  |  | 00000037 | repeat region | 3 |
|  |  |  |  | 00000043 | repeat region | 3 |
|  |  |  |  | 00000001 | repeat region | 2 |
|  |  |  |  | 00000002 | repeat region | 2 |
|  |  |  |  | 00000003 | repeat region | 2 |
|  |  |  |  | 00000005 | repeat region | 2 |
|  |  |  |  | 00000011 | repeat region | 2 |
|  |  |  |  | 00000013 | repeat region | 2 |
|  |  |  |  | 00000014 | repeat region | 2 |
|  |  |  |  | 00000033 | repeat region | 2 |
|  |  |  |  | 00000036 | repeat region | 2 |
|  |  |  |  | 00000046 | repeat region | 2 |
|  |  |  |  | 00001380 INT | Copia LTR retrotransposon | 2 |
|  |  |  |  | 00002725 INT | Copia LTR retrotransposon | 2 |
|  |  |  |  | 00000000 | repeat region | 1 |
|  |  |  |  | 00000006 | repeat region | 1 |
|  |  |  |  | 00000018 | repeat region | 1 |
|  |  |  |  | 00000019 | repeat region | 1 |
|  |  |  |  | 00000020 | repeat region | 1 |
|  |  |  |  | 00000024 | repeat region | 1 |
|  |  |  |  | 00000025 | repeat region | 1 |
|  |  |  |  | 00000026 | repeat region | 1 |
|  |  |  |  | 00000028 | repeat region | 1 |
|  |  |  |  | 00000029 | repeat region | 1 |
|  |  |  |  | 00000031 | repeat region | 1 |
|  |  |  |  | 00000041 | repeat region | 1 |
|  |  |  |  | 00000042 | repeat region | 1 |
|  |  |  |  | 00000047 | repeat region | 1 |
|  |  |  |  | 00000049 | repeat region | 1 |
|  |  |  |  | 00000051 | repeat region | 1 |
|  |  |  |  | 00000054 | repeat region | 1 |
|  |  |  |  | 00000056 | repeat region | 1 |
|  |  |  |  | 00000174 | repeat region | 1 |
|  |  |  |  | 00000426 | repeat region | 1 |
|  |  |  |  | 00000486 | repeat region | 1 |
|  |  |  |  | 00001187 | repeat region | 1 |
|  |  |  |  | 00001226 | Mutator TIR transposon | 1 |
|  |  |  |  | 00001228 | helitron | 1 |
|  |  |  |  | 00001279 | helitron | 1 |
|  |  |  |  | 00001280 | helitron | 1 |
|  |  |  |  | 00001313 | helitron | 1 |
|  |  |  |  | 00001325 | Mutator TIR transposon | 1 |
|  |  |  |  | 00001326 | helitron | 1 |
|  |  |  |  | 00001431 | helitron | 1 |
|  |  |  |  | 00001438 | helitron | 1 |
|  |  |  |  | 00001467 | Tc1 Mariner TIR transposon | 1 |
|  |  |  |  | 00001525 | CACTA TIR transposon | 1 |
|  |  |  |  | 00001935 INT | LTR retrotransposon | 1 |
|  |  |  |  | 00002067 | hAT TIR transposon | 1 |
|  |  |  |  | 00002686 | CACTA TIR transposon | 1 |
|  |  |  |  | 00003180 INT | LTR retrotransposon | 1 |
|  |  |  |  | 00003536 | hAT TIR transposon | 1 |
|  |  |  |  | 00003567 | Mutator TIR transposon | 1 |
|  |  |  |  | 00003771 | Tc1 Mariner TIR transposon | 1 |
|  |  |  |  | 00003853 | Mutator TIR transposon | 1 |
| 1b | 1 | 292,191-331,870 | 39,680 | 00000189 | repeat region | 4 |
|  |  |  |  | 00000032 | repeat region | 3 |
|  |  |  |  | 00000005 | repeat region | 2 |
|  |  |  |  | 00000013 | repeat region | 2 |
|  |  |  |  | 00000016 | repeat region | 2 |
|  |  |  |  | 00000024 | repeat region | 2 |
|  |  |  |  | 00001454 INT | Gypsy LTR retrotransposon | 2 |
|  |  |  |  | 00000000 | repeat region | 1 |
|  |  |  |  | 00000019 | repeat region | 1 |
|  |  |  |  | 00000028 | repeat region | 1 |
|  |  |  |  | 00000029 | repeat region | 1 |
|  |  |  |  | 00000048 | repeat region | 1 |
|  |  |  |  | 00001213 | Mutator TIR transposon | 1 |
|  |  |  |  | 00001226 | Mutator TIR transposon | 1 |
|  |  |  |  | 00001325 | Mutator TIR transposon | 1 |
|  |  |  |  | 00001467 | Tc1 Mariner TIR transposon | 1 |
|  |  |  |  | 00002725 INT | Copia LTR retrotransposon | 1 |
| 2 | 2 | 709-244,868 | 244,160 | 00001485 | CACTA TIR transposon | 28 |
|  |  |  |  | 00003603 | Mutator TIR transposon | 28 |
|  |  |  |  | 00000007 | repeat region | 27 |
|  |  |  |  | 00000012 | repeat region | 25 |
| 3a | 3 | 313-45,415 | 45,103 | 00003603 | Mutator TIR transposon | 13 |
|  |  |  |  | 00000192 | repeat region | 6 |
|  |  |  |  | 00002527 LTR | Gypsy LTR retrotransposon | 2 |
| 3b | 3 | 70,362-194,455 | 124,094 | 00002320 | helitron | 36 |
|  |  |  |  | 00000007 | repeat region | 12 |
|  |  |  |  | 00001485 | CACTA TIR transposon | 12 |
|  |  |  |  | 00000129 | repeat region | 9 |
|  |  |  |  | 00002130 | helitron | 9 |
|  |  |  |  | 00000012 | repeat region | 8 |
|  |  |  |  | 00001328 | PIF Harbinger TIR transposon | 7 |
|  |  |  |  | 00003603 | Mutator TIR transposon | 5 |
|  |  |  |  | 00003622 INT | Gypsy LTR retrotransposon | 5 |
|  |  |  |  | 00001574 | Mutator TIR transposon | 1 |
| 4a | 4 | 744-494,348 | 493,605 | 00003603 | Mutator TIR transposon | 118 |
|  |  |  |  | 00000027 | repeat region | 3 |
|  |  |  |  | 00000037 | repeat region | 3 |
|  |  |  |  | 00000043 | repeat region | 3 |
|  |  |  |  | 00000001 | repeat region | 2 |
|  |  |  |  | 00000011 | repeat region | 2 |
|  |  |  |  | 00000033 | repeat region | 2 |
|  |  |  |  | 00000036 | repeat region | 2 |
|  |  |  |  | 00001380 INT | Copia LTR retrotransposon | 2 |
|  |  |  |  | 00001454 INT | Gypsy LTR retrotransposon | 2 |
|  |  |  |  | 00000003 | repeat region | 1 |
|  |  |  |  | 00000018 | repeat region | 1 |
|  |  |  |  | 00000020 | repeat region | 1 |
|  |  |  |  | 00000041 | repeat region | 1 |
|  |  |  |  | 00000042 | repeat region | 1 |
|  |  |  |  | 00000047 | repeat region | 1 |
|  |  |  |  | 00000051 | repeat region | 1 |
|  |  |  |  | 00000426 | repeat region | 1 |
|  |  |  |  | 00001187 | repeat region | 1 |
|  |  |  |  | 00001279 | helitron | 1 |
|  |  |  |  | 00001313 | helitron | 1 |
|  |  |  |  | 00001326 | helitron | 1 |
|  |  |  |  | 00001431 | helitron | 1 |
|  |  |  |  | 00002067 | hAT TIR transposon | 1 |
|  |  |  |  | 00002725 INT | Copia LTR retrotransposon | 1 |
|  |  |  |  | 00003536 | hAT TIR transposon | 1 |
| 4b | 4 | 494,349-1,118,478 | 624,130 | 00003603 | Mutator TIR transposon | 4 |
|  |  |  |  | 00000222 | repeat region | 1 |
| 4c | 4 | 1,265,318-2,153,008 | 887,691 | 00000007 | repeat region | 164 |
|  |  |  |  | 00001485 | CACTA TIR transposon | 164 |
|  |  |  |  | 00003603 | Mutator TIR transposon | 88 |
|  |  |  |  | 00003622 INT | Gypsy LTR retrotransposon | 46 |
|  |  |  |  | 00001728 | hAT TIR transposon | 1 |
| 4d | 4 | 2,153,009-2,283,220 | 130,212 | 00003603 | Mutator TIR transposon | 26 |
|  |  |  |  | 00000007 | repeat region | 17 |
|  |  |  |  | 00001485 | CACTA TIR transposon | 17 |
|  |  |  |  | 00000192 | repeat region | 6 |
|  |  |  |  | 00003622 INT | Gypsy LTR retrotransposon | 6 |
|  |  |  |  | 00002320 | helitron | 4 |
|  |  |  |  | 00002527 LTR | Gypsy LTR retrotransposon | 4 |
|  |  |  |  | 00000012 | repeat region | 2 |
|  |  |  |  | 00000129 | repeat region | 1 |
|  |  |  |  | 00001611 LTR | Gypsy LTR retrotransposon | 1 |
|  |  |  |  | 00002130 | helitron | 1 |
|  |  |  |  | 00003983 LTR | LTR retrotransposon | 1 |
| 4e | 4 | 2,283,221-3,423,733 | 1,140,513 | 00003603 | Mutator TIR transposon | 1 |
| 4f | 4 | 3,728,810-3,788,149 | 59,340 | 00003603 | Mutator TIR transposon | 17 |
|  |  |  |  | 00000192 | repeat region | 6 |
|  |  |  |  | 00002527 LTR | Gypsy LTR retrotransposon | 6 |
|  |  |  |  | 00003983 LTR | LTR retrotransposon | 1 |
| 5 | 5 | 47,990,044-48,117,911 | 127,868 | 00000189 | repeat region | 13 |
|  |  |  |  | 00000032 | repeat region | 6 |
|  |  |  |  | 00001454 INT | Gypsy LTR retrotransposon | 5 |
|  |  |  |  | 00003603 | Mutator TIR transposon | 5 |
|  |  |  |  | 00000005 | repeat region | 4 |
|  |  |  |  | 00000013 | repeat region | 4 |
|  |  |  |  | 00000016 | repeat region | 4 |
|  |  |  |  | 00000024 | repeat region | 4 |
|  |  |  |  | 00000007 | repeat region | 3 |
|  |  |  |  | 00000017 | repeat region | 3 |
|  |  |  |  | 00001485 | CACTA TIR transposon | 3 |
|  |  |  |  | 00002725 INT | Copia LTR retrotransposon | 3 |
|  |  |  |  | 00000000 | repeat region | 2 |
|  |  |  |  | 00000001 | repeat region | 2 |
|  |  |  |  | 00000003 | repeat region | 2 |
|  |  |  |  | 00000019 | repeat region | 2 |
|  |  |  |  | 00000028 | repeat region | 2 |
|  |  |  |  | 00000029 | repeat region | 2 |
|  |  |  |  | 00000031 | repeat region | 2 |
|  |  |  |  | 00000046 | repeat region | 2 |
|  |  |  |  | 00000048 | repeat region | 2 |
|  |  |  |  | 00000052 | repeat region | 2 |
|  |  |  |  | 00001213 | Mutator TIR transposon | 2 |
|  |  |  |  | 00001226 | Mutator TIR transposon | 2 |
|  |  |  |  | 00001280 | helitron | 2 |
|  |  |  |  | 00001289 | Tc1 Mariner TIR transposon | 2 |
|  |  |  |  | 00001325 | Mutator TIR transposon | 2 |
|  |  |  |  | 00001467 | Tc1 Mariner TIR transposon | 2 |
|  |  |  |  | 00001525 | CACTA TIR transposon | 2 |
|  |  |  |  | 00002686 | CACTA TIR transposon | 2 |
|  |  |  |  | 00003567 | Mutator TIR transposon | 2 |
|  |  |  |  | 00000006 | repeat region | 1 |
|  |  |  |  | 00000009 | repeat region | 1 |
|  |  |  |  | 00000012 | repeat region | 1 |
|  |  |  |  | 00000022 | repeat region | 1 |
|  |  |  |  | 00000041 | repeat region | 1 |
|  |  |  |  | 00000042 | repeat region | 1 |
|  |  |  |  | 00000047 | repeat region | 1 |
|  |  |  |  | 00000057 | repeat region | 1 |
|  |  |  |  | 00000426 | repeat region | 1 |
|  |  |  |  | 00001228 | helitron | 1 |
|  |  |  |  | 00001326 | helitron | 1 |
|  |  |  |  | 00002181 | Mutator TIR transposon | 1 |
| 6 | 6 | 11,619,789-11,664,202 | 44,414 | 00002782 INT | Gypsy LTR retrotransposon | 13 |
|  |  |  |  | 00000189 | repeat region | 10 |
|  |  |  |  | 00000017 | repeat region | 5 |
|  |  |  |  | 00000002 | repeat region | 4 |
|  |  |  |  | 00001454 INT | Gypsy LTR retrotransposon | 4 |
|  |  |  |  | 00000005 | repeat region | 2 |
|  |  |  |  | 00000013 | repeat region | 2 |
|  |  |  |  | 00000014 | repeat region | 2 |
|  |  |  |  | 00000174 | repeat region | 2 |
|  |  |  |  | 00000486 | repeat region | 2 |
|  |  |  |  | 00000000 | repeat region | 1 |
|  |  |  |  | 00000025 | repeat region | 1 |
|  |  |  |  | 00000026 | repeat region | 1 |
|  |  |  |  | 00000028 | repeat region | 1 |
|  |  |  |  | 00000031 | repeat region | 1 |
|  |  |  |  | 00000049 | repeat region | 1 |
|  |  |  |  | 00000054 | repeat region | 1 |
|  |  |  |  | 00000056 | repeat region | 1 |
|  |  |  |  | 00001226 | Mutator TIR transposon | 1 |
|  |  |  |  | 00001280 | helitron | 1 |
|  |  |  |  | 00001467 | Tc1 Mariner TIR transposon | 1 |
|  |  |  |  | 00001525 | CACTA TIR transposon | 1 |
|  |  |  |  | 00002686 | CACTA TIR transposon | 1 |
|  |  |  |  | 00002725 INT | Copia LTR retrotransposon | 1 |
|  |  |  |  | 00003567 | Mutator TIR transposon | 1 |
|  |  |  |  | 00003771 | Tc1 Mariner TIR transposon | 1 |
|  |  |  |  | 00003853 | Mutator TIR transposon | 1 |
| 9 | 9 | 61,518,546-61,627,824 | 109,279 | 00000189 | repeat region | 9 |
|  |  |  |  | 00002782 INT | Gypsy LTR retrotransposon | 7 |
|  |  |  |  | 00000032 | repeat region | 6 |
|  |  |  |  | 00000017 | repeat region | 3 |
|  |  |  |  | 00001454 INT | Gypsy LTR retrotransposon | 3 |
|  |  |  |  | 00000002 | repeat region | 2 |
|  |  |  |  | 00000005 | repeat region | 2 |
|  |  |  |  | 00000013 | repeat region | 2 |
|  |  |  |  | 00000016 | repeat region | 2 |
|  |  |  |  | 00000024 | repeat region | 2 |
|  |  |  |  | 00000048 | repeat region | 2 |
|  |  |  |  | 00000000 | repeat region | 1 |
|  |  |  |  | 00000019 | repeat region | 1 |
|  |  |  |  | 00000025 | repeat region | 1 |
|  |  |  |  | 00000026 | repeat region | 1 |
|  |  |  |  | 00000028 | repeat region | 1 |
|  |  |  |  | 00000029 | repeat region | 1 |
|  |  |  |  | 00000031 | repeat region | 1 |
|  |  |  |  | 00000056 | repeat region | 1 |
|  |  |  |  | 00000174 | repeat region | 1 |
|  |  |  |  | 00000486 | repeat region | 1 |
|  |  |  |  | 00001213 | Mutator TIR transposon | 1 |
|  |  |  |  | 00001226 | Mutator TIR transposon | 1 |
|  |  |  |  | 00001280 | helitron | 1 |
|  |  |  |  | 00001325 | Mutator TIR transposon | 1 |
|  |  |  |  | 00001467 | Tc1 Mariner TIR transposon | 1 |
|  |  |  |  | 00001525 | CACTA TIR transposon | 1 |
|  |  |  |  | 00002552 | PIF Harbinger TIR transposon | 1 |
|  |  |  |  | 00002686 | CACTA TIR transposon | 1 |
|  |  |  |  | 00002725 INT | Copia LTR retrotransposon | 1 |
|  |  |  |  | 00003567 | Mutator TIR transposon | 1 |
|  |  |  |  | 00003853 | Mutator TIR transposon | 1 |

**Supplementary Table 8.** The number of contigs in diverse wild species accessions used in Tang *et al.* (2022) with >80% homology to the unanchored-contig clustered regions (UCCRs 1a–9) found in the *S. bulbocastanum* genome, revealing that most UCCRs were rare except for 1a, 1b, 5, and 6.

| UCCR  identity | *S. palustre*  PG0009 | *S. etuberosum*  PG0019 | *S. morelliforme*  PG1011 | *S. pinnatisectum*  PG1013 | *S. jamesii*  PG1008 | *S. bulbocastanum*  PG6241 | *S. andreanum*  PG3003 | *S. piurae*  PG3023 |
| --- | --- | --- | --- | --- | --- | --- | --- | --- |
| 1a | 0 | 1 | 0 | 0 | 8 | 0 | 5 | 1 |
| 1b | 4 | 5 | 37 | 24 | 27 | 8 | 51 | 8 |
| 2 | 0 | 0 | 0 | 0 | 0 | 0 | 0 | 0 |
| 3a | 0 | 0 | 0 | 0 | 0 | 1 | 0 | 0 |
| 3b | 0 | 0 | 0 | 0 | 0 | 0 | 0 | 0 |
| 4a | 0 | 0 | 0 | 0 | 0 | 0 | 0 | 0 |
| 4b | 0 | 0 | 0 | 0 | 0 | 51 | 0 | 0 |
| 4c | 0 | 0 | 0 | 0 | 0 | 0 | 0 | 0 |
| 4d | 0 | 0 | 0 | 0 | 0 | 0 | 0 | 0 |
| 4e | 0 | 0 | 0 | 0 | 0 | 33 | 0 | 0 |
| 4f | 0 | 0 | 0 | 0 | 0 | 0 | 0 | 0 |
| 5 | 0 | 3 | 0 | 0 | 0 | 0 | 5 | 0 |
| 6 | 8 | 6 | 40 | 14 | 32 | 4 | 41 | 8 |
| 9 | 0 | 1 | 0 | 0 | 0 | 0 | 0 | 0 |

**Supplementary Table 8.** Continued.

| UCCR  identity | *S. multiinterruptum*  PG4060 | *S. cajamarquense*  PG6242 | *S. burkartii*  PG4005 | *S. chomatophilum*  PG3005 | *S. sogarandinum*  PG4032 | *S. boliviense*  PG5076 | *S. commersonii*  PG4049 |
| --- | --- | --- | --- | --- | --- | --- | --- |
| 1a | 2 | 15 | 12 | 2 | 20 | 4 | 17 |
| 1b | 51 | 51 | 51 | 51 | 51 | 51 | 51 |
| 2 | 0 | 0 | 0 | 0 | 0 | 0 | 0 |
| 3a | 0 | 0 | 0 | 0 | 0 | 0 | 0 |
| 3b | 0 | 0 | 0 | 0 | 0 | 0 | 0 |
| 4a | 0 | 0 | 0 | 0 | 0 | 0 | 0 |
| 4b | 0 | 0 | 0 | 0 | 0 | 0 | 0 |
| 4c | 0 | 0 | 0 | 0 | 0 | 0 | 0 |
| 4d | 0 | 0 | 0 | 0 | 0 | 0 | 0 |
| 4e | 0 | 0 | 0 | 0 | 0 | 0 | 0 |
| 4f | 0 | 0 | 0 | 0 | 0 | 0 | 0 |
| 5 | 0 | 4 | 4 | 0 | 9 | 3 | 11 |
| 6 | 49 | 51 | 51 | 48 | 51 | 51 | 51 |
| 9 | 0 | 0 | 0 | 0 | 0 | 0 | 0 |

**Supplementary Table 8.** Continued.

| UCCR  identity | *S. chacoense*  PG4042 | *S. paucissectum*  PG3022 | *S. brevicaule*  PG5032 | *S. neorossii*  PG6243 | *S. vernei*  PG4036 | *S. buesii*  PG4041 | *S. lignicaule*  PG4017 | *S. multidissectum*  PG5068 |
| --- | --- | --- | --- | --- | --- | --- | --- | --- |
| 1a | 1 | 7 | 3 | 2 | 2 | 14 | 0 | 19 |
| 1b | 51 | 51 | 51 | 48 | 51 | 51 | 51 | 51 |
| 2 | 2 | 0 | 0 | 0 | 0 | 0 | 0 | 1 |
| 3a | 0 | 0 | 0 | 0 | 0 | 0 | 0 | 0 |
| 3b | 0 | 0 | 0 | 0 | 0 | 0 | 0 | 0 |
| 4a | 0 | 0 | 0 | 0 | 0 | 0 | 0 | 0 |
| 4b | 0 | 0 | 0 | 0 | 0 | 0 | 0 | 0 |
| 4c | 0 | 0 | 0 | 0 | 0 | 0 | 0 | 0 |
| 4d | 0 | 0 | 0 | 0 | 0 | 0 | 0 | 0 |
| 4e | 0 | 0 | 0 | 0 | 0 | 0 | 0 | 0 |
| 4f | 0 | 0 | 0 | 0 | 0 | 0 | 0 | 0 |
| 5 | 0 | 0 | 3 | 0 | 0 | 5 | 0 | 14 |
| 6 | 49 | 51 | 51 | 26 | 50 | 51 | 40 | 51 |
| 9 | 0 | 0 | 0 | 0 | 0 | 0 | 0 | 0 |

**Supplementary Table 8.** Continued.

| UCCR  identity | *S. stenotomum*  PG6029 | *S. stenotomum*  PG6359 | *S. stenotomum*  A6-26 | *S. phureja*  E86-69 | *S. phureja*  E4-63 | 2*x* *S. tuberosum*  RH10-15 | Total |
| --- | --- | --- | --- | --- | --- | --- | --- |
| 1a | 0 | 0 | 0 | 0 | 1 | 0 | 136 |
| 1b | 4 | 12 | 6 | 9 | 33 | 34 | 1024 |
| 2 | 0 | 0 | 0 | 0 | 0 | 0 | 3 |
| 3a | 0 | 0 | 0 | 0 | 0 | 0 | 1 |
| 3b | 0 | 0 | 0 | 0 | 0 | 0 | 0 |
| 4a | 0 | 0 | 0 | 0 | 0 | 0 | 0 |
| 4b | 0 | 0 | 0 | 0 | 0 | 0 | 51 |
| 4c | 0 | 0 | 0 | 0 | 0 | 0 | 0 |
| 4d | 0 | 0 | 0 | 0 | 0 | 0 | 0 |
| 4e | 0 | 0 | 0 | 0 | 0 | 0 | 33 |
| 4f | 0 | 0 | 0 | 0 | 0 | 0 | 0 |
| 5 | 0 | 0 | 0 | 0 | 0 | 1 | 62 |
| 6 | 2 | 6 | 1 | 7 | 12 | 17 | 919 |
| 9 | 0 | 0 | 0 | 0 | 0 | 1 | 2 |

**Supplementary Table 9.** The number of homologous regions (>80%) to the unanchored-contig clustered regions (UCCRs 1a–9) found in the *S. bulbocastanum* genome in chromosome-scale assemblies of related species, revealing that the UCCRs are limited to the *S. bulbocastanum* genome except for a few cases.

| UCCR identity | *S. phureja* | *S. multidissectum* | *S. chacoense* | *S. verrucosum* | *S. bulbocastanum* | *S. etuberosum* | *S. lycopersicum* |
| --- | --- | --- | --- | --- | --- | --- | --- |
| 1a | 0 | 0 | 0 | 0 | 1 | 0 | 1 |
| 1b | 0 | 0 | 0 | 0 | 5 | 0 | 2 |
| 2 | 0 | 3 | 0 | 0 | 1 | 0 | 0 |
| 3a | 0 | 0 | 0 | 0 | 3 | 0 | 0 |
| 3b | 0 | 0 | 0 | 0 | 1 | 0 | 0 |
| 4a | 0 | 0 | 0 | 0 | 1 | 0 | 0 |
| 4b | 0 | 0 | 0 | 0 | 1 | 0 | 0 |
| 4c | 0 | 0 | 0 | 0 | 1 | 0 | 0 |
| 4d | 0 | 0 | 0 | 0 | 1 | 0 | 0 |
| 4e | 0 | 0 | 0 | 0 | 1 | 0 | 0 |
| 4f | 0 | 0 | 0 | 0 | 1 | 0 | 0 |
| 5 | 0 | 0 | 0 | 0 | 1 | 0 | 0 |
| 6 | 0 | 0 | 0 | 0 | 1 | 0 | 1 |
| 9 | 0 | 0 | 0 | 0 | 1 | 0 | 1 |
